# Seasonal variation in metabolic profiles and microbial communities in a subarctic ore processing plant

**DOI:** 10.1111/1758-2229.13284

**Published:** 2024-06-23

**Authors:** Malin Bomberg, Hanna Miettinen, Päivi Kinnunen

**Affiliations:** ^1^ VTT Technical Research Centre of Finland Ltd. Espoo Finland; ^2^ VTT Technical Research Centre of Finland Ltd. Tampere Finland

## Abstract

The mining industry strives to reduce its water footprint by recycling water in ore processing. This leads to build‐up of ions, flotation chemicals and microbial biomass, which may affect the process. The Boliden Kevitsa mine in Northern Finland is exposed to seasonal change and recycles up to 90% of the process water. We studied the variation in size, composition and putative functions of microbial communities in summer and winter in the ore processing plant. The raw water, Cu and Ni thickener overflow waters had statistically significantly higher bacterial numbers in winter compared to summer, and specific summer and winter communities were identified. Metagenomic analysis indicated that Cu and Hg resistance genes, sulphate/thiosulphate, molybdate, iron(III) and zinc ABC transporters, nitrate reduction, denitrification, thiosulphate oxidation and methylotrophy were more common in winter than in summer. Raw water drawn from the nearby river did not affect the microbial communities in the process samples, indicating that the microbial communities and metabolic capacities develop within the process over time in response to the conditions in the processing plant, water chemistry, used chemicals, ore properties and seasonal variation. We propose that the microbial community structures are unique to the Boliden Kevitsa mine and processing plant.

## Graphical abstract

The microbial communities in a northern ore processing plant were compared in summer and winter. Distinct seasonal communities were identified with higher microbial numbers and specific metabolic capacities, such as heavy metal resistance, sulphate/thiosulphate, molybdate, iron(III) and Zinc transport, nitrate reduction, denitrification, thiosulphate oxidation and methylotrophy more common in winter.

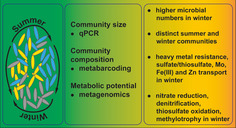

## INTRODUCTION

The mining industry is responsible for an estimated 1–2% of the annual global freshwater use (Kalin et al., 2018) and can in dry areas take up to one order of magnitude more (Aitken et al., 2016; Alvez et al., 2020; Askham & Van der Poll, 2017; Department of Water and Sanitation, 2016; Dieter et al., 2018; Valdés‐Pineda et al., 2014). Most of this water is used in flotation and concentration processes (Gunson et al., 2012; Li et al., 2019). The managing of outflowing process water (PW) also adds to the general expenses of the mine and ore processing plant because used PW needs to be extensively cleaned before release into the environment. A key solution to make the metals and raw materials industry more sustainable and decrease its environmental footprint is to recycle and reuse the PW, which in turn decreases the need for water intake and effluent release.

Recycling of PW may have both positive and negative effects on the flotation processes in the ore processing plant and adds complexity (Kinnunen et al., 2021). In addition to the decreased need for intake of fresh water, the residual flotation reagents used may decrease the need for adding more reagents. However, build‐up of flotation reagents and microorganisms may also cause unspecific flotation of metals, which may decrease or increase the grade of the concentrate (Miettinen et al., 2023; Muzinda & Schreithofer, 2018). Moreover, recycling may cause build‐up of different ions, such as Ca^2+^, Na^+^, Mg^2+^, K^+^ and SO_4_^2−^, which may then precipitate as, for example, magnesium carbonate or gypsum, which may form scaling and clogging in the PW circuit and contaminate mineral surfaces (Bulut & Yenial, 2016; Muzinda & Schreithofer, 2018).

Although the physicochemical challenges with recycling of PW may be known and predictable, only little is thus far known about the role of microbes in flotation processes (Kinnunen et al., 2020; Miettinen et al., 2023). Microorganisms are present in natural water and in ore and will be introduced into the flotation processes through the intake water and ore and are also deposited from air and precipitation. The build‐up of organic flotation chemicals, such as xanthates and carboxymethyl cellulose (CMC), may also cause an increase in microbial numbers in the recycled water, because the microorganisms may use these compounds as carbon and energy sources. In addition, residual nitrogenous compounds, especially nitrate, originating from blasting reagents may serve as nitrogen source or electron acceptor for the microbial communities. The ore itself may also provide nutrients, such as sulphur compounds, iron compounds and carbonates, which can be used by the microorganisms and also serve as drivers to form specific microbial community compositions in different parts of the water circuit.

The microbial load may be greatly affected by the seasons. Annual day length was shown to be one of the main factors affecting the microbial community composition in the English Channel water, where the diversity of non‐phototrophic microorganisms peaked around the shortest day lengths of approximately 8 h of daylight (Gilbert et al., 2012). Monitoring of the microbial communities over the year showed that in the River Rhine water, the highest microbial numbers were observed in winter and spring (Bergfeld et al., 2011), whereas in the surface water of the alpine lake Gossenköllesee, Austria, the highest number of microbial cells were detected in August–September and January (Pernthaler et al., 1998). In the Boliden Kevitsa mine flotation process, the microbial community was monitored over a 2‐month period of August and September (Bomberg et al., 2020). It was found that in PW the number of bacterial 16S rRNA gene copies fluctuated between 3.3 × 10^5^ ml^−1^ in August and 5.1 × 10^4^ ml^−1^ in September, when the air and water temperatures decreased.

**FIGURE 1:**
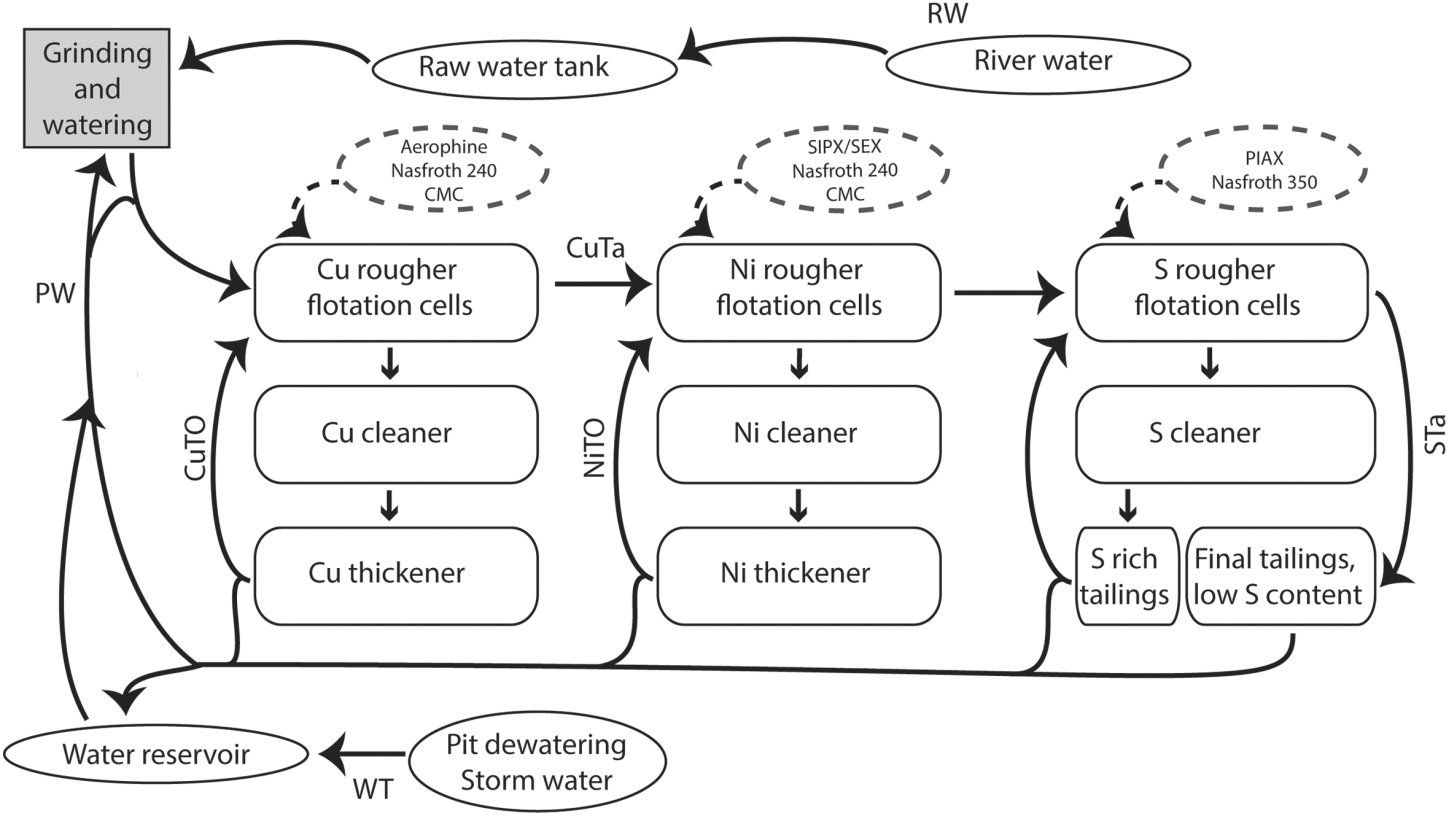
Simplified outline of the mineral processing plant of the Boliden Kevitsa mine. The flow of water is indicated by arrows, boxes indicate different compartments of the flotation plant, ovals indicate water sources and the dashed ovals and arrows show the flotation chemicals used in the different processes (Aerophine – phosphine‐based collector, Nasfroth 240 and 350 – polyglycol ether frother, CMC – Carboxymethyl cellulose depressant, SIPX – Sodium Isopropyl Xanthate, SEX – Sodium Ethyl Xanthate, PIAX – Potassium Isoamyl Xanthate). The sampling points are indicated by sample codes (RW – river water; WT – water reservoir/tank; PW – process water, that is, water cycling through the plant; CuTA – tailings from the Cu flotation; STa – tailings from the S flotation; CuTO – overflow from the Cu thickener; NiTO – overflow from the Ni thickener).

The Boliden Kevitsa mine is a multi‐metal mine situated in the north of Finland (Berthet, 2020; Kokko et al., 2014). The mine produces, among other metals, copper and nickel, and has its own processing plant close to the open mine pit. The ore processing includes crushing and grinding of the ore, separate Cu, Ni and S flotation circuits and concrete dewatering circuits (Figure 1). The Boliden Kevitsa mine recycles approximately 90% of the PW used in the plant (Le et al., 2021; Muzinda & Schreithofer, 2018). The Boliden Kevitsa mine processing plant location north of Arctic circle with midnight sun and polar night as well as temperatures ranging from over 20°C to below −20°C experiences seasonal variations in precipitation and water quality, which may affect the flotation performance (Le et al., 2021; Muzinda & Schreithofer, 2018) as well as biological aspects. The aim of this study was to investigate the seasonal effects (summer vs. winter) on the chemical parameters and microbiological community composition and metabolic capacities in different parts of the plant water circuit.

## EXPERIMENTAL PROCEDURES

### Sampling site

The Boliden Kevitsa mine processing plant contains ore crushing and grinding compartments and indoors flotation and concrete dewatering circuits. Intake fresh water originates from the nearby Vajukoski river. Water from the different processes as well as pit dewatering and storm water streams are collected into a water reservoir for reuse.

### Samples for chemistry and microbiology

Water samples for chemical analyses were collected on two occasions in 2018 on February 5th in order to study the winter effect and on August 28th for the summer effect. Samples were collected from river water (RW), water tank (WT), PW, copper flotation tailings (CuTa), sulphur flotation tailings (STa), copper and nickel thickener overflow (CuTO and NiTO, respectively) (Table S1). The samples for chemical analyses were first collected into clean buckets and temperature (T,°C), pH, dissolved oxygen (mg L^−1^), pH, specific conductance (SPCond. μS cm^−1^), conductance (Cond. μS cm^−1^) and oxidation–reduction potential (mV) were measured immediately using a YSI ProDSS‐110 multisensor probe (YSI Inc., Yellow Springs, OH, USA). For further chemical analyses, solids were removed from the sample water by filtration using 1.6 μm pore‐size filter paper and vacuum suction. Samples for cation analysis were further filtered using 0.45 μm pore‐size syringe filters into HNO_3_ rinsed bottles. Samples for total and dissolved organic carbon (TOC and DOC, respectively) and total carbon (TC) were filtered through 1.2 μm pore‐size filters into 100 ml plastic bottles. Sample for thiosulphate, sulphate, bromide, chloride, nitrate, nitrite, nitrate‐N and nitrite‐N were filtered using 1.2 μm pore‐size filters into plastic bottles and were further filtered with 0.45 μm pore‐size filters at the analysis laboratory. Samples for total nitrogen, ammonium, ammonium‐N, phosphate and phosphate‐P were filtered with a 1.2 μm pore‐size filters into plastic bottles. All samples were cooled and sent for analysis to Eurofins Lahti, Finland, and analysed according to the protocols of the service provider (Table 1).

**TABLE 1:** The physicochemical parameters measured from the different samples during the summer and winter campaign. The pH, T, EC and ORP measurements were done on site using a pH probe. All other measurements were done by Eurofins Lahti according to their methods.

	Summer							Winter						
	RW	WT	PW	CuTa	STa	CuTO	NiTO	RW	WT	PW	CuTa	STa	CuTO	NiTO
pH	7.3	8.0	7.2	9.7	9.3	9.4	9.2	7.0	9.3	8.9	9.9	9.7	10.8	8.6
EC, μS cm^−1^	36	3085	2660	2637	2639	1979	2450	50	1451	2837	2660	2730	2311	2630
T,°C	19	8	12	23	23	16	14	7.8	0.8	1.9	12.3	12.4	6.3	4.5
ORP, mV/SHE	391	352	205	202	225	259	245	383	272	199	169	202	264	296
TDS, g L^−1^	0.04	2.9	1.9	2.1	2.0	1.4	1.7	0.04	0.47	1.9	2.2	2.0	1.5	1.6
TOC, mg L^−1^	7	7	12	13	20	14	13	6.5	7	19	19	22	18	19
DOC, mg L^−1^	6.3	6.1	12	13	20	13	13	6.3	6.6	18	19	23	18	19
TIC, mg L^−1^	2.7	36	7.5	2.1	1.8	3.9	7.6	2.6	43	4.3	2.4	2.3	2.8	5.5
TC, mg L^−1^	10	43	20	16	23	18	22	9.6	52	23	23	26	22	25
Thiosalts^a^, mg L^−1^	2.3	1500	740	1900	4200	900	730	2.4	130	990	1100	1200	1100	1100
Thiosulphate, mg L^−1^	<5.0	<25	<10	58	44	23	11	<5.0	<5.0	55	73	70	72	53
Sulphate, mg L^−1^	2.4	1500	700	600	660	520	720	2.2	110	690	530	620	530	610
S dissolved, mg L^−1^	0.95	510	260	310	300	200	260	0.84	46	240	210	280	240	250
N_tot_, mg L^−1^	0.3^b^	36	5.1	3.9	3.7	3.2	6.6	0.4^b^	32	4.5	5.7	3.8	4.3	6.0
Nitrate, mg L^−1^	<1	140	9.4	8.4	9.7	6.8	19	<1	41	13	14	13	9.5	14
Nitrate‐N, mg L^−1^	<0.25	31	2.1	1.9	2.2	1.5	4.2	<0.25	9.3	2.9	3.1	3	2.2	3.1
Nitrite‐N, mg L^−1^	<35	82	75	76	67	67	130	<35	1400	190	200	210	150	210
Ammonium‐N, mg L^−1^	<0.004	0.15	1.1	1.1	1.1	0.86	1.1	<0.004	22	2.2	2.5	2.3	1.9	2.3
Cl, mg L^−1^	2.2	130	420	570	520	290	340	5	61	470	640	580	350	420
Br, mg L^−1^	<0.05	1.2	2.6	3.4	3	1.9	2.2	<0.05	0.6	3.3	3.9	3.8	2.5	2.7
K, mg L^−1^	1.6	48	49	54	52	33	43	4.4	23	61	66	57	46	53
Ca, mg L^−1^	2.8	180	150	150	140	110	150	3	9.2	160	180	160	210	160
Mg, mg L^−1^	1.1	300	70	54	61	44	67	1.1	54	92	61	74	20	83
Na, mg L^−1^	1.3	43	190	260	240	130	150	1.4	19	230	350	270	170	200
Si, mg L^−1^	1.8	7.3	11	9.8	11	5.8	7.4	2.3	6.9	8.2	8.9	10	2.9	7.2
As, μg L^−1^	<1.0	<1.0	2.9	<1.0	<1.0	<1.0	1.4	<1.0	7.8	2.4	3.8	6.7	<1.0	2.5
Ba, μg L^−1^	26	51	130	190	180	69	85	4.3	14	97	290	160	84	83
P, μg L^−1^	23	43	120	160	130	86	110	<10	<10	130	150	110	130	140
Cu, μg L^−1^	12	15	15	<3.0	<3.0	7.7	8.8	3.6	3.3	<3.0	<3.0	<3.0	<3.0	<3.0
Mn, μg L^−1^	6.3	200	53	<5.0	<5.0	<5.0	34	9.9	6.5	23	<5.0	6.6	<5.0	25
Ni, μg L^−1^	<1.0	1400	140	24	28	76	130	<1.0	30	62	19	32	10	91
Fe, μg L^−1^	400	<50	270	<50	<50	<50	<50	540	67	<50	<50	270	<50	<50
Zn, μg L^−1^	28	7.8	19	<5.0	6.8	<5.0	<5.0	<5.0	<5.0	<5.0	<5.0	<5.0	<5.0	<5.0
Sr, μg L^−1^	11	290	460	590	550	330	420	12	89	530	680	550	450	470

^a^ Sum of thiosulphate, thiosulphite and sulphate.

^b^ CFA method.

Sample water and slurries for microbiology were collected into sterile 1 L or 250 ml plastic bottles (Nalgene, Rochester, NY, USA). Of the water samples subsamples between 250 and 500 ml were filtered in triplicate on 0.22 μm pore‐size Sterivex polyethersulphone (Merck Millipore, Burlington, MA, USA) syringe filter units using sterile 100 ml syringes (KD‐JECT III, KD Medical, Columbia, MD, USA) after which the filter units were placed in sterile 50 ml plastic test tubes equipped with screw caps (BD Falcon, BD, Franklin Lakes, NJ, USA) and frozen at −20°C. Slurry samples were frozen directly at −20°C. All samples were shipped frozen in coolers to the VTT laboratory, Espoo, Southern Finland for further processing.

### DNA extraction

The Sterivex filter units containing the microbial biomass were thawed in their storage tubes on ice and thereafter moved to sterile plastic bags, which were protected with aluminium foil layers, and carefully smashed open with a hammer. The aluminium foil‐plastic bag packages were carefully opened in a laminar flow hood avoiding contamination and the filters were cut from the central cylinder of the filtration unit using sterile scalpels and placed in 5‐ml centrifugation tubes (Eppendorf, Hamburg, Germany) for DNA extraction. The slurry samples were carefully defrosted at +4°C and were then mixed thoroughly. From the homogeneous sample, 5 ml aliquots were placed in 5 ml Eppendorf tubes (Eppendorf) and centrifuged on an Eppendorf 5010R table‐top centrifuge at 3184*g* for 10 min in order to pellet microorganisms and solid material. The supernatant was discarded, and the pellet was subjected to DNA extraction. The DNA was extracted with the NucleoSpin Soil DNA extraction kit (Macherey‐Nagel, Düren, Germany) using the beads from two lysis tubes and double volumes of the lysis buffer SL1 and enhancer solution SX. Thereafter the DNA extraction procedure followed the manufacturer's instructions, and the DNA was eluted in 100 μl (Sterivex samples) or 60 μl (slurry samples) SE buffer. The DNA extracts were further purified using the NucleoSpin gDNA Clean‐up kit (Macherey‐Nagel) according to the manufacturer's protocol and eluted in the original (100 μl or 60 μl) volume.

For slurry samples that produce low DNA yield with commercial DNA extraction kits, a new method for extracting DNA using phenol and anionic nanocellulose was developed and used for DNA extraction from samples CuTa and STa summer and winter samples as previously described (Bomberg & Miettinen, 2023). Samples for which this method was used will hereafter be indicated with an F, that is, CuTaF and STaF. For DNA extraction, three replicate slurry samples varying between 27 and 32.5 ml were used from each sample (Table S1). Negative reagent controls without sample material were processed in parallel with the samples.

### Estimation of community size by qPCR

The number of bacterial and archaeal 16S rRNA genes and eukaryotic 5.8S rRNA genes were estimated by qPCR as described in Bomberg et al. (2020) and Bomberg and Miettinen (2020). The bacterial communities were targeted with primers Bact_0341F/Bact_805R (Herlemann et al., 2011) in triplicate 10 μl reactions using the SensiFAST SYBR No‐ROX 2 × master mix for LightCycler480 (Bioline, London, UK). The archaeal communities were targeted with primers A344F (Bano et al., 2004) and A744R modified from Barns et al. (1994) and the detection was performed using the archaeal specific A516P probe equipped with an FAM‐label (Bomberg & Miettinen, 2020). The eukaryotes were targeted using a probe‐based qPCR approach (Haugland & Vesper, 2002) using the primers 5.8F1 and 5.8R1 and detected with an FAM‐labelled probe 5.8P1. The archaeal and eukaryotic assays were performed with the SensiFAST Probe No‐ROX kit (Bioline) in 10 μl reaction volumes and triplicate reactions for each sample.

### Amplicon sequencing

Amplicon libraries of bacterial and archaeal 16S rRNA genes and eukaryotic ITS1 fragments for sequencing on the Ion torrent PGM (ThermoFisher Scientific, Waltham, MA, USA) were produced as described in Bomberg et al. (2020). The bacterial amplicon libraries were produced with primers Bact_0341F/Bact_805R (Herlemann et al., 2011), the archaeal amplicon libraries with primers S‐D‐Arch‐0349‐a‐S‐17/S‐D‐Arch‐0787‐a‐A‐20 (Klindworth et al., 2013) and the eukaryotic communities with primer pair ITS1 and ITS2 (Gardes & Bruns, 1993; White et al., 1990). Parallel 25 μl amplification reactions were prepared for each sample in 1x MyTaq Red Mix (Bioline), with 800 nM of each primer, up to 25 μl molecular‐biology‐grade water (Sigma‐Aldrich, Saint Louis, MO, USA) and 2 μl of template. The PCR program contained an initial denaturation step at 95°C for 3 min, followed by 35 cycles for bacteria and eukaryotes and 40 cycles for archaea of 15 s denaturation at 95°C, 15 s primer annealing at 50°C, and 15 s elongation at 72°C, and a final 30 s elongation step at 72°C. The PCR products were verified with agarose gel electrophoresis before they were sent for sequencing on the Ion Torrent PGM platform at Bioser (Oulu, Finland). The amplicons were further purified and size‐selected before sequencing by Bioser.

### Amplicon sequence analysis

The sequence reads were analysed using the Mothur software (v.1.43.0) (Schloss et al., 2009) as described in Bomberg et al. (2020). Operational Taxonomic Units (OTUs) of the bacterial and archaeal sequences were determined at the 97% sequence homology threshold and the taxonomy of the OTUs were assigned based on the Silva database version 138_1 (Quast et al., 2012; Yilmaz et al., 2014). The eukaryotic ITS1 sequence reads were also grouped into OTUs by VSEARCH clustering of the unaligned sequences using an internal OTU sequence similarity of 97% as described in Bomberg et al. (2020). The OTUs were classified using the Unite version 8.2 ITS database covering all eukaryotes as reference (dated 2020‐02‐04, UNITEv8_sh_dynamic_s_all) (Kõljalg et al., 2020; Nilsson et al., 2019). Sequences that were not classified as bacteria, archaea or eukaryotes in the respective sequence data sets were removed.

The analysed sequence data was extracted as biom tables for subsequent alpha‐ and betadiversity analyses.

The sequences have been submitted to the European Nucleotide Archive (http://www.ebi.ac.uk/ena) under study accession number PRJEB67656.

### Statistical analyses

Only samples with more than 200 sequence reads were included in the statistical analyses. Alpha diversity analyses, including the Shannon diversity indices and the estimated Chao and ACE OTU richness of the amplicon data were performed with Phyloseq (McMurdie & Holmes, 2013) in R (Computing, 2013). Principal coordinate analysis (PCoA) on the similarities between the community compositions between the different samples was calculated using Phyloseq in R. The data used in the PCoA analyses was relative abundance of OTUs and Bray–Curtis dissimilarity. One‐way ANOVA in the PAST 3.0 package (Hammer et al., 2008) was used to test the differences in the number of bacterial and archaeal 16S and eukaryotic 5.8S rRNA genes between the samples and between seasons. The correlation between physicochemical parameters and relative abundance of the most common, that is, >1% relative abundance in at least one sample, bacterial genera based on amplicon sequence data, or the predicted abundance of genes according to the detection level of the genes (how many times a gene was completely covered by the sequence read data) in the metagenomic data was calculated with PAST 3.0. Multivariate analysis of variance (MANOVA) was used to analyse the co‐occurrence of different metabolic pathways and processes and was calculated using PAST 3.0.

### Metagenomic sequencing

DNA from RW, WT, PW, CuTO and NiTO summer and winter samples were subjected to metagenomic analysis. Equal amounts of DNA from all three replicate samples were combined and used as template for multiple displacement amplification using the Illustra Genomi Phi V2 (GE Healthcare) amplification kit according to the manufacturer's instructions. Two parallel amplification reactions were performed for each sample's DNA mixture. For each reaction, 2 μl sample DNA was mixed with 18 μl sample buffer in 250 μl PCR tubes and heated at 95°C for 3 min. Thereafter, the reactions were cooled on cooling blocks (Eppendorf) and 18 μl reaction buffer and 2 μl Phi29 polymerase solution was added to each reaction. The amplification reactions were incubated for 2 h at 30°C in an Eppendorf MasterCycler (Eppendorf) and the reaction was terminated by heating to 65°C for 1 min, after which the reactions were cooled at 4°C. The amplified DNA from each reaction was measured using the Qubit fluorometric quantification assay (ThermoFisher Scientific) for dsDNA. The parallel amplification reactions from each sample were combined and purified using the NucleoSpin gDNA Clean‐up kit (Macherey‐Nagel) and eluted in 50 μl elution buffer. The DNA amounts were checked using the Qubit assay and the quality of the DNA was examined using the Nanodrop 1000 spectrophotometer (Thermo Scientific, Waltham, MA, USA). The amplified DNA samples of 0.5 μg in 50 μl were sent to Eurofins Genomics (Konstanz, Germany) for metagenomic sequencing using the Illumina platform and 2 × 150 bp sequencing library preparation.

### Metagenomic analyses

A detailed description of the metagenomic analyses is presented in Supplement A. Briefly, the quality of the sequence data was checked using Fastqc (Andrews, 2010), merged using SeqPrep (St John, 2011) and trimmed using Trimmomatic (Bolger et al., 2014). The sequence data of all samples was co‐assembled using the MEGAHIT assembler (Li et al., 2015). The trimmed sequence reads were separately mapped to the co‐assembly using Bowtie2 (Langdon, 2015). The data was further analysed using anvi'o v. 6.1 (Eren et al., 2015), and anvi'o identified gene calls were taxonomically annotated using Kaiju v 1.7.3 using the nr_euk database version 2019‐06‐25 (Menzel et al., 2016) and the taxonomical classifications were imported to anvi'o.

Functional annotations were done against the ncbi COGs database and Pfams (El‐Gebali et al., 2019), PROKKA version (Seemann, 2014) and GHOSTKOALA (Kanehisa et al., 2016).

Gene coverages and detection in the tested samples was extracted from anvi'o and the metagenomic data is presented as detection frequencies, which indicate how deeply (i.e., the minimum layer of sequence reads at any point of a gene) a specific gene was completely covered by mapping the sequence reads to the contigs.

## RESULTS

### Chemistry

The pH of the different samples varied between 7.3 and 9.7 in the summer and 7.0 and 10.8 in the winter (Table 1). The water temperature was 8–23°C and 0.8–12.4°C, electrical conductivity (EC) 36–3085 and 50–2837 mS cm^−1^, and Redox 202–391 and 199–383 mV/SHE in the summer and winter samples, respectively (Table 1). The concentration of TOC and DOC were especially high in the plant samples, with the exception of the WT. In addition, the organic carbon concentration was generally higher in the winter samples, compared to the summer samples. The RW and WT contained between 6.1 and 6.6 mg L^−1^ organic carbon (TOC and DOC) without differences between the summer and winter samples. The concentration of total inorganic carbon (TIC) was not generally different in the summer and winter samples, or between the RW samples and the plant samples and had concentrations between 1.8 and 7.6 mg L^−1^. Nevertheless, the WT sample differed from the rest by containing TIC concentrations of 36 and 43 mg L^−1^ in the summer and winter samples, respectively. This inconsistency was also visible in the measurement of TC. The concentration of thiosalts was between 730 and 4200 mg L^−1^ in the plant samples in summer, and lower, between 130 and 1200 mg L^−1^ in winter and consisted mostly of sulphate. The total concentration of nitrogen (N_tot_) was between 0.3 and 0.4 mg L^−1^ in the RW sample in summer and winter, respectively, and 10–22 times higher in the plant samples, with the exception of the WT sample, where the N_tot_ reached 36 and 32 mg L^−1^ in summer and winter, respectively. The nitrate‐N and ammonium‐N were the greatest contributing sources of the N_tot_. Phosphate‐P was detectable only in the RW samples and in the summer WT sample at concentrations of 15–26 μg L^−1^ in summer and 2.7 μg L^−1^ in winter. In all other samples, the phosphate‐P content was below the detection limit of the assay. However, the concentration of soluble P was between 86 and 160 μg L^−1^ in all plant samples, with exception of the WT. The RW and WT samples had 23 and 43 μg L^−1^ P in the summer sample, but less than 10 μg L^−1^ in the winter samples. Compared to the RW, elevated concentrations of Cl, Na, Br, K, Ca, Mg, Si, S and Sr, were detected in the plant samples, with generally higher concentrations in winter, compared to the summer samples. S and Sr were exceptions, with higher concentrations measured in the summer. Cu was detected at low concentrations in all summer samples, except the CuTa and STa tailings samples but was below the detection limit in the winter samples, with the exception of the WT. The RW contained 12 and 3.3 μg L^−1^ Cu in summer and winter, respectively. The concentration of Ni was below the detection limit of 1 μg L^−1^ in the RW samples. In the plant samples, the Ni concentration varied between 19 and 1400 μg L^−1^, with generally higher concentrations seen in the summer samples. Zn was only detected in the summer samples. The highest Zn concentration was measured in the RW, whereas the concentration was lower in the WT, PW and STa, and below detection limit in the rest of the samples.

### Microbial populations

**FIGURE 2:**
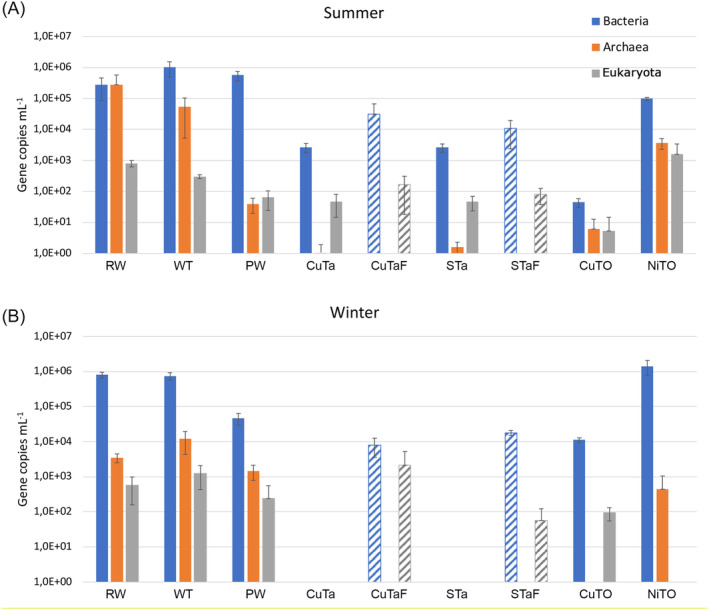
The number of bacterial (blue) and archaeal (orange) 16S rRNA gene copies and eukaryotic (grey) 5.8S rRNA gene copies ml^−1^ in the (A) summer and (B) winter samples. The striped columns of CuTaF and STaF indicate the phenol‐extraction protocol. Each column is the average of three replicate qPCR reactions from two or three replicate samples (*n* = 6–9) and the error bars show standard deviation.

The amount of DNA obtained from the different samples is presented in Table SA2. Bacterial 16S rRNA gene copy numbers were detected between 2.7 × 10^3^ and 1.0 × 10^6^ ml^−1^ in the summer samples, with the exception of only 4.4 × 10^1^ bacterial genes ml^−1^ detected from sample CuTO. The bacterial 16S rRNA gene copy numbers in the winter samples varied between 1.2 × 10^4^ and 1.4 × 10^6^ ml^−1^. However, no bacterial 16S rRNA genes were detected by qPCR from the CuTa and STa winter samples (Figure 2). According to Mann–Whitney's Bonferroni corrected pairwise test, the number of bacterial 16S rRNA genes ml^−1^ was statistically significantly higher (*p* < 0.05) in the winter than in the summer in the RW, CuTO and NiTO, but not in the other sampling points. However, Dunn's post hoc test with Bonferroni correction did not reveal statistically significant differences between the seasons. The customized DNA extraction protocol provided one order of magnitude higher number of bacterial 16S rRNA genes for the summer CuTaF and STaF compared to CuTa and STa prepared with the commercial DNA extraction kit. Furthermore, whereas no bacterial 16S rRNA gene copies were detected by qPCR from CuTa and STa winter samples, the bacterial 16S rRNA genes numbers were 8.0 × 10^3^ and 1.8 × 10^4^ ml^−1^, for CuTaF and STaF, respectively.

Archaeal 16S rRNA genes were detected at 4.0 × 10^1^–2.8 × 10^5^ copies ml^−1^ in RW, WT, PW and NiTO in the summer samples and between 4.5 × 10^2^ and 1.2 × 10^4^ ml^−1^ in the winter (Figure 2). In all other samples, the number of archaeal 16S rRNA genes ml^−1^ was below 10^1^ or below the detection limit of the sample. Only sample NiTO showed statistically significant difference between the summer and winter samples (*p* < 0.05) according to the Bonferroni corrected Mann–Whitney test, but not according to Dunn's post hoc test. The phenol‐protocol did not affect the number of detected archaeal 16S rRNA genes.

Eukaryotic 5.8S rRNA genes were detected at the level of 4.8 × 10^1^–1.6 × 10^3^ copies ml^−1^ in most of the summer samples, with the exception of CuTO (Figure 2). In the winter samples, the level of eukaryotic 5.8S rRNA genes was 9.4 × 10^1^–1.2 × 10^3^ ml^−1^ in RW, WT, PW and CuTO, but not detected from the CuTa and STa winter samples. Nevertheless, eukaryotic 5.8S rRNA genes were detected from the CuTaF and STaF samples, both in the summer (1.6 × 10^2^ and 8.1 × 10^1^ copies, respectively), and in the winter (2.2 × 10^3^ and 5.7 × 10^1^ copies ml^−1^, respectively).

The average number of bacterial sequence reads obtained from the samples extracted with the NucleoSpin Soil DNA extraction kit and resulting in more than 200 sequence reads (31 out of 42 samples) was 6.3 × 10^3^ ± 5.2 × 10^3^, and from the phenol‐extracted samples (CuTaF, STaF) with more than 200 sequence reads (11 out of 12 samples) 1.7 × 10^3^ ± 8.5 × 10^2^ (Table SA3). Samples with less than 200 sequence reads were omitted from the rest of the analyses. The average number of bacterial OTUs varied between 25 and 1137, the Chao1 and ACE estimated OTU richness was between 31 and 1479 and 44 and 1292, respectively. The Shannon diversity index varied between 1.8 and 4.7.

**FIGURE 3:**
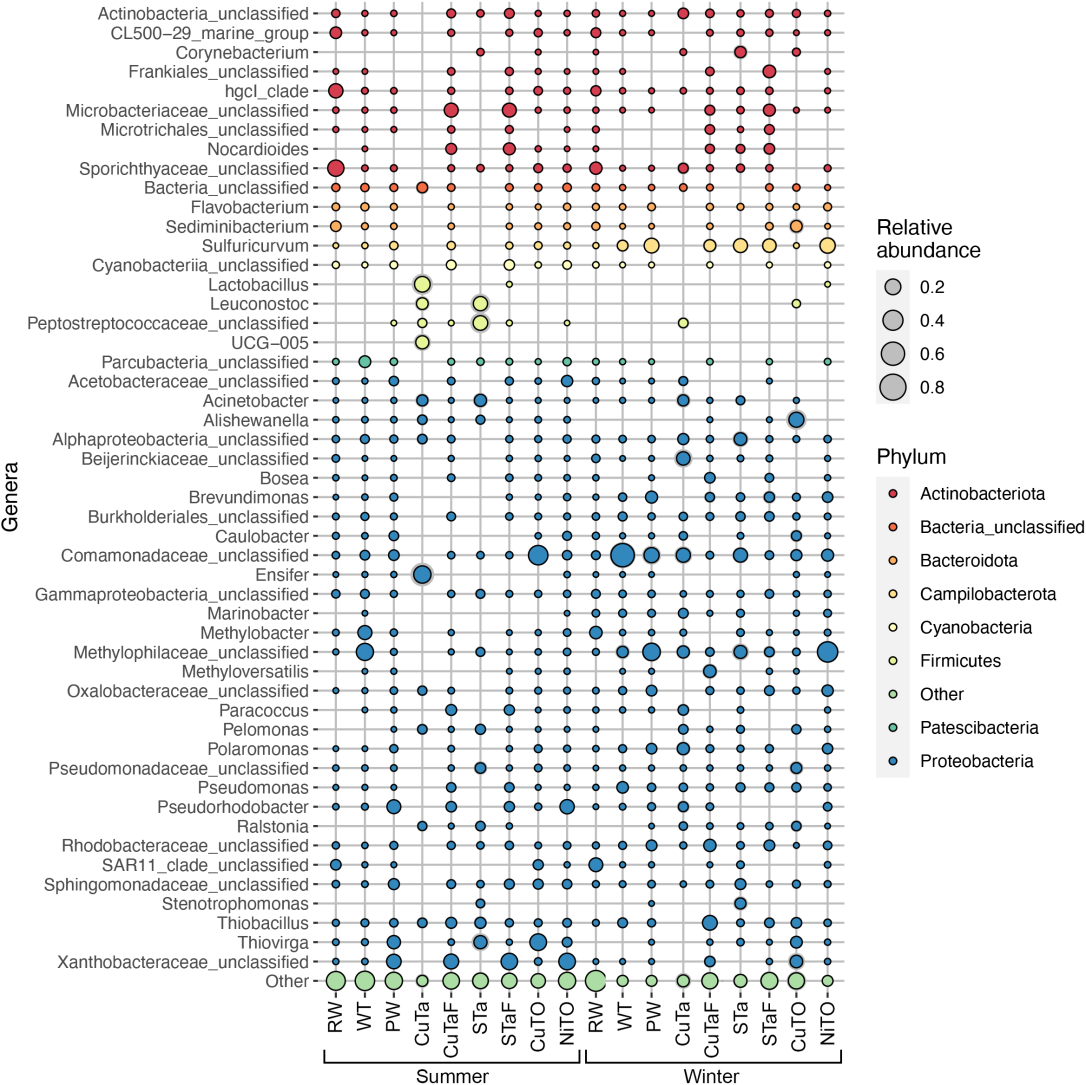
The average relative abundance of the 50 most prevalent bacterial genera in the summer and winter samples based on three replicate samples, with the exception of summer CuTa, for which bacterial 16S rRNA gene sequence data was obtained from only two replicate samples. “Other” indicates all other less abundant genera.

Proteobacteria was the most commonly detected bacterial phylum, contributing with 16.4–98.1% of the total bacterial sequence reads in the samples (Figure 3, Figure SA1, Figure SA2). The bacterial sequence reads constituted to 32.1–52.0% of Alphaproteobacteria in the summer PW, CuTaF, STaF and NiTO samples (Figure SA2). In these samples, unclassified *Xanthobacteraceae* were the most common alphaproteobacterial clade (Supplement B) present in all four sample types. *Pseudorhodobacter* were also common in the PW and NiTO summer samples, but not in the winter samples. SAR_11 clade alphaproteobacteria were present in all RW samples and they were more prominent in the winter, and also in the CuTO summer sample, although not generally detected in any other samples.

The highest proportion of gammaproteobacterial sequence reads was obtained from the WT summer and winter samples, PW winter, CuTO summer and NiTO winter samples (Supplement B, Figure 3, Figure SA1, Figure SA3). Unclassified Methylophiliaceae were the most common gammaproteobacterial clade in the summer WT and winter PW and NiTO, whereas in the CuTO summer and WT winter, the most common gammaproteobacteria belonged to an unclassified genus of the Commamonadaceae. In addition, *Thiovirga* was detected in the PW and CuTO samples in the summer, but not in the winter. *Thiobacillus* was especially well detected in CuTaF, both in the summer and winter.

The RW bacterial community composition differed between summer and winter (Supplement B). The dominating bacteria in the summer were unclassified Sporichthyaceae, unclassified Ilumatobacteraceae and hgcI clade (19.5–24.8%, 6.2–7.4% and 13.9–16.5% of the bacterial community, respectively) of the Actinobacteriota, *Sediminibacter* (4.7–5.0%) of the Bacteroidota and unclassified SAR11 clade (3.8–5.2%) of the Alphaproteobacteria. Except for the SAR11, the proportion of these bacteria decreased in the winter. The winter community was dominated by SAR11 (11.4–16.9%), unclassified Sporichthyaceae (5.2–10.9%) and *Methylobacter* (8.8–9.7%).

The WT bacterial community was dominated by methylotrophic and nitrate reducing bacteria in the summer, with genera belonging to the Methylophiliaceae and Methylomonadaceae contributing with 25.0–29.0% and 13.7–15.6% of the bacterial community, respectively (Supplement B). In addition, sequence reads belonging to the Patescibacteria (*Candidatus* Kaiserbacteria, *Candidatus* Nomurabacteria and unclassified Parcubacteria) made up 9.5–18.3% of the winter community. The winter community was dominated by unclassified Comamonadaceae bacteria (37.3–76.5%).

The PW contained sequence reads of unclassified Xanthobacteraceae (15.3–16.1%), *Pseudorhodobacter* (12.1–15.6%) and *Thiovirga* (11.3–11.7%) in the summer, but in the winter the community consisted mostly of *Sulfuricurvum* (11.2–19.9%), *Brevundimonas* (5.9–10.0%), unclassified Commamonadaceae (7.7–37.8%) and unclassified Methylococcaceae (15.3–33.9%).

A higher number of sequence reads were obtained from CuTaF and STaF than from the CuTa and STa samples. In the CuTaF the majority of the bacterial community consisted of unclassified Microbacteriaceae (10.8–15.7%), unclassified Xanthobacteraceae (18.3–20.6%) and *Thiobacillus* (3.7–7.12%), whereas the winter community was dominated by *Sulfuricurvum* (2.3–15.9%), unclassified Rhodobacteraceae (6.7–11.9%), *Thiobacillus* (12.4–20.7%), *Methyloversatilis* (up to 19.0%), unclassified Xanthobacteraceae (up to 10.5%) and unclassified Planococcaceae (up to 8.6%). The STaF summer community consisted mostly of unclassified Xanthobacteraceae (13.8–30.0%), unclassified Microbacteriaceae (5.7–20.8%) and Nocardioides (up to 10.1%). The STaF winter community was different, with unclassified Frankiales (up to 14.1%) and *Sulfuricurvum* (up to 20.5%), although unclassified Microbacteriaceae (up to 12.4%) and Nocardioides (up to 10.9%) were also found.

In the CuTO summer samples, unclassified Commamonadaceae and *Thiovirga* were the most common bacteria contributing with 35.2–39.5% and 20.3–28.2% of the bacterial community, respectively. Only one of the three replicate winter CuTO samples provided enough sequences to be included in the analysis. This sample was dominated by *Shewanella* (47.8%), *Thiovirga* (11.0%) and unclassified Pseudomonadaceae (16.3%).

The NiTO summer bacterial population contained unclassified Xanthomonadaceae and *Pseudorhodobacter* (21.0–26.4% and 13.2–16.5%. respectively), whereas the winter community consisted of *Sulfuricurvum* (12.2–29.0%), unclassified Commamonadaceae (7.1–8.8%) and unclassified Methylophiliaceae (29.9–47.9%).

Altogether 946 different bacterial genera were identified from the different samples (Supplement C).

More than 200 archaeal 16S rRNA gene sequence reads were obtained from only a small number of samples (Table SA4). In general, archaeal sequences were only obtained from all three replicate samples of RW, and from the winter WT and PW samples. However, the number of observed OTUs, Chao1 and ACE as well as the Shannon diversity index was quite high (Table SA4).

**FIGURE 4:**
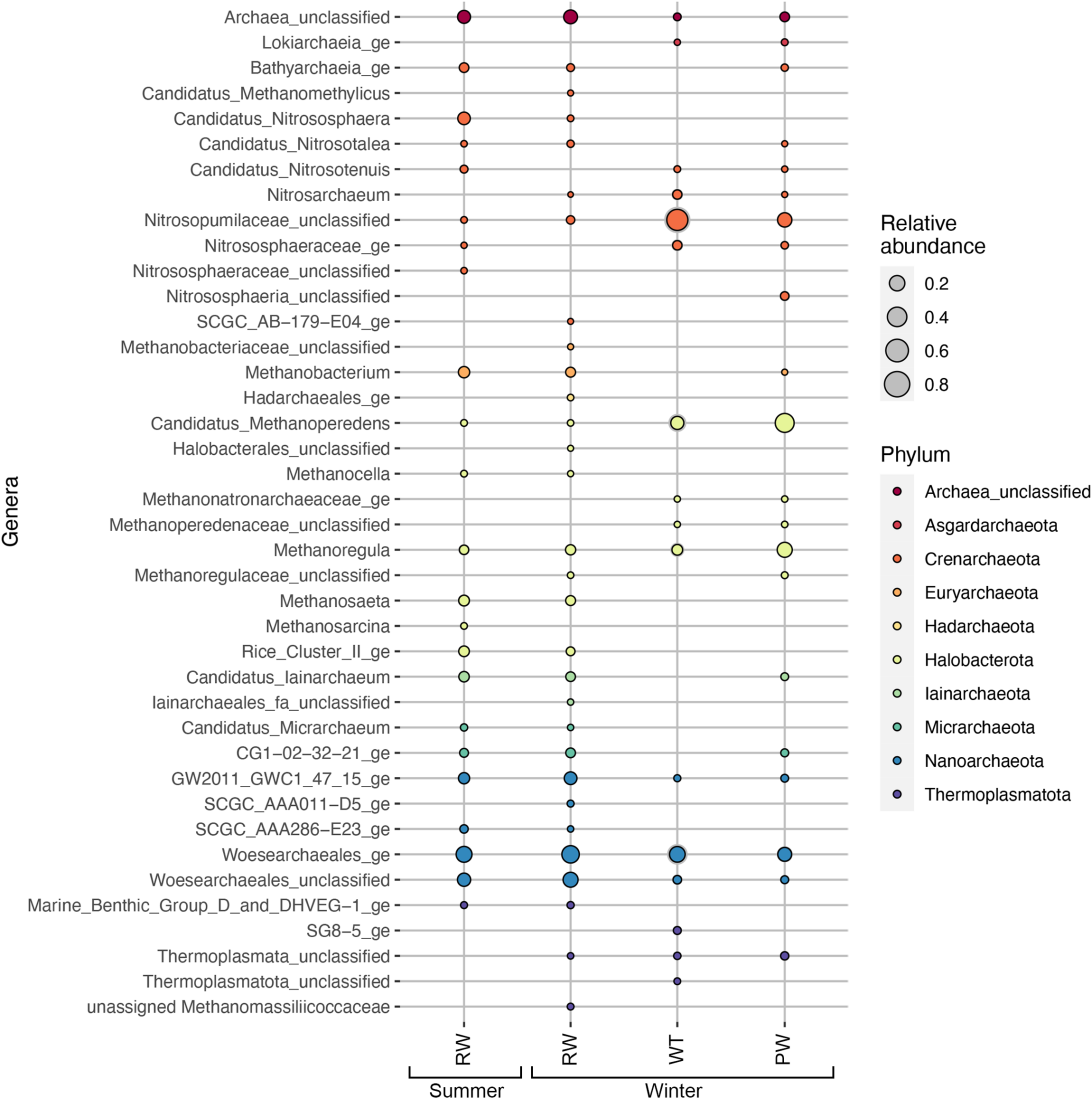
The average relative abundances of archaeal genera detected in the samples. Adequate numbers of archaeal sequence reads were obtained only from the summer RW (three replicate samples), and winter RW (three samples), WT (one sample) and PW (two samples).

In the RW samples most of the archaeal community was represented by Nanoarchaeota sequences belonging to the order Woesearchaeales, 42.5–78.1% (Figure 4, Figure SA4). The Woesearchaeales sequence reads were divided onto three main genera, the GW2011_GWC1_47_15_ge, Woesearchaeales_ge and unclassified Woesearchaeales, each contributing with 4.7–28.9% of the sequence reads. *Candidatus* Nitrososphaera were more prominent in the summer, contributing with 8.5–10.1% of the archaeal communities, but were below 0.5% in the winter samples. Bathyarchaeales were present at relative abundances of 2.5–3.5% in the summer but were less frequent in winter.

The archaeal sequence reads in the summer and winter RW consisted of up to 9.1, 8.8, 6.2, 6.3 and 17.9% of *Methanobacteria*, *Methanoregula*, Rice Cluster II archaea, *Methanosaeta*, *Candidatus* Iainarchaeum and unclassified Archaea. In the winter PW sample, up to 20.2, 20.6, 43.2 and 21.8 and 6.2% of the archaeal sequence belonged to unclassified Nitrosopumilaceae, *Methanoregula*, *Candidatus* Methanoperedens, Woesearchaeales_ge and unclassified Archaea, respectively. In all other sample types, the number of archaeal sequence reads was too low, and the samples were excluded.

More than 200 eukaryotic ITS1 sequence reads were obtained from 32 out of 42 samples submitted to the NucleoSpin Soil DNA extraction protocol, with an average sequence read number of 1.5 × 10^3^ ± 1.8 × 10^3^ (Table SA5). The average sequence read number of the phenol‐extracted samples (11 samples out of 12 with more than 200 sequence reads) was 1.8 × 10^3^ ± 9.5 × 10^2^. The number of eukaryotic OTUs identified from the samples varied between 5 and 265 (Table SA5) and the Chao1 and ACE estimated OUT richness was 4–333 and 5–331, respectively. The Shannon's diversity index was as low as 0.08 in one CuTa winter sample and as high as 3.9 in the summer WT sample.

**FIGURE 5:**
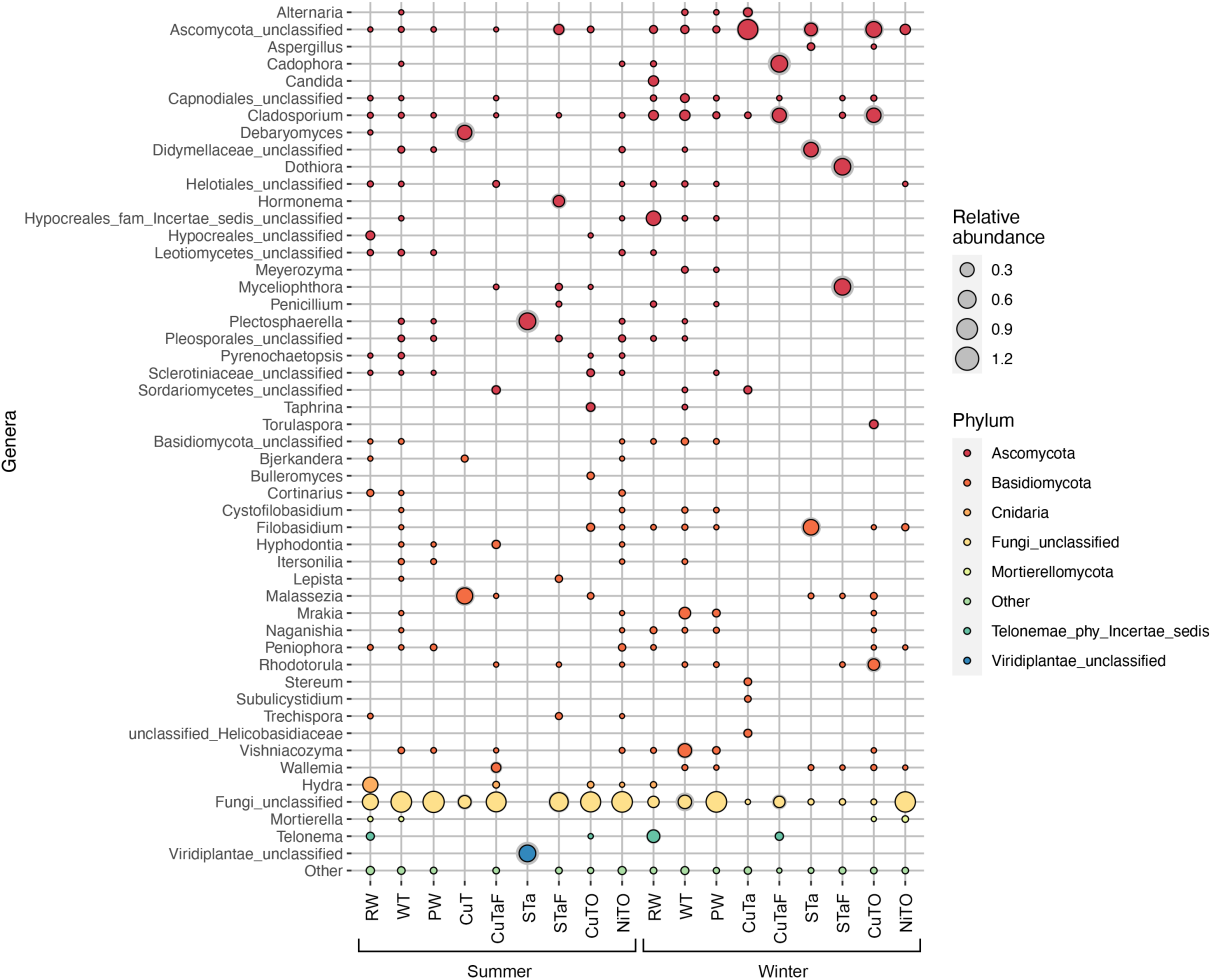
The average relative abundance of the 50 most prominent eukaryotic genera in the samples. Each sampling point is represented by three replicate samples, with the exception of summer STa, and winter CuTaF and STaF, for which eukaryotic ITS sequences were obtained from only two replicate samples. “Other” indicates all other less abundant genera.

Most of the eukaryotic communities consisted of fungi that remained without specific identification (fungi unclassified). In the summer samples, from which eukaryotes were detected, unclassified fungi dominated the eukaryotic communities with 35.0–97.8% of the eukaryotic sequence reads. In the winter samples, unclassified fungi were prominent in the PW and NiTO samples, representing 71.3–97.5% of the eukaryotic communities. Of the identified fungal phyla, Ascomycota were represented by 1.5–99.7% of the eukaryotic sequence reads, while Basidiomycota with 0–85% of the eukaryotic sequence reads overall. Ascomycota were more prevalent in the winter samples and especially in CuTa, STa and CuTO (Figure 5, Figure SA5). Basidiomycota were more sporadically detected. Cnidaria (*Hydra* sp.) were found in the summer RW samples but were not present elsewhere. Unclassified Hypocreales were the most common Ascomycete fungi in the summer RW samples, contributing with 3.3–7.8% of the eukaryotic sequence reads but were mostly absent in all other samples. The winter RW samples contained a different Hypocreales group falling with the Hypocreales *Incertae sedis* and contributing with 21.3–42.9% of the eukaryotic sequence reads. This group was present at very low relative abundance or remained undetected in the other samples. Both summer and winter RW samples contained a high relative abundance of *Telonema*, which were not detected in the process samples, except for one winter CuTaF sample, which had 7.8% *Telonema* sequences. The winter RW contained *Cladosporium* (2.7–12.6%) and *Candida* (8.6–11.5%). *Cladosporium* was also found (1.7–18.2%) in all winter WT samples, but only sporadically in other process samples, whereas *Candida* was not found in any process samples. Instead, the winter WT sample had 2.3–9.9% unclassified Capnodiales (Ascomycota) and 9.5–27.1% and 3.5–47.6% *Mrakia* and *Vishniacozyma*, respectively, all of which were also detected in one PW sample, but only sporadically and at very low relative abundance from the process samples. Otherwise unclassified Ascomycota were the most commonly detected eukaryotic sequence in most winter process samples.

### Principal coordinate analysis

**FIGURE 6:**
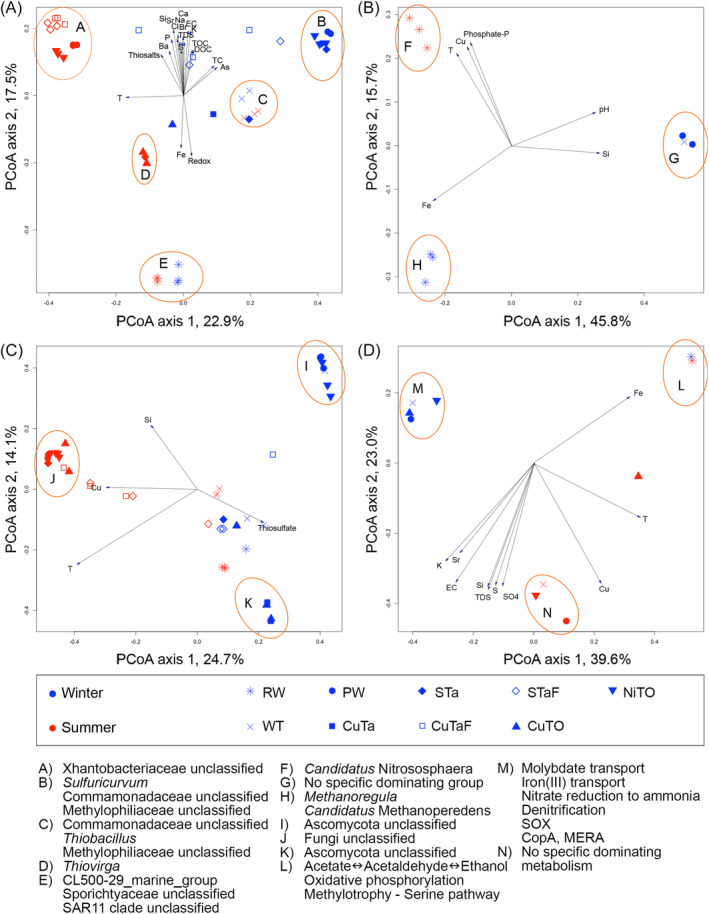
Principal coordinate analysis (PCoA) based on the Bray–Curtis dissimilarity model of the (A) bacterial, (B) archaeal and (C) eukaryotic communities characterized by amplicon sequencing using relative abundance of OTUs and (D) the abundance of genes based on the detection frequency (i.e., the average depth of coverage by sequence reads over the whole gene) of the predicted genes in the metagenomic data. The environmental parameters with statistical significance of (A) *p* < 0.001, (B) *p* < 0.01, (C) *p* < 0.05 and (D) *p* < 0.05 are shown by the vectors.

The PCoA showed that in all other cases but for the eukaryotes, the RW samples were clearly different from the samples collected from the different sites of the process plant (Figure 6). In addition, the bacterial and eukaryotic communities as well as the metagenomic profiles were similar in summer and winter RW samples, whereas the archaeal communities were highly dissimilar.

With the exception of the WT samples, the bacterial communities of the process samples were roughly separated into summer communities in the middle and upper left of the PCoA plot, where temperature had the strongest influence of the community, and winter communities in the middle and upper right, where the TC and As had the strongest influence (Figure 6A). The summer and winter WT bacterial communities clustered closely together, indicating that the bacterial communities of the WT did not change between seasons. In the upper left corner, the phenol‐extracted summer CuTaF and STaF communities formed a tight cluster with the summer PW and NiTO sample communities, whereas the single commercial kit extracted STa sample from which sequence reads were obtained fell with CuTO samples. This indicates that the bacterial community present in the water circuit binds strongly to the solids present and could only be detached and detected with harsh DNA extraction method. The winter CuTaF and STaF communities were strongly influenced by the highest concentrations of all measured chemical parameters, except Fe. The CuTaF and STaF communities profiles also fell separately from the CuTa and STa communities.

Archaea were mainly detected in the RW, summer and winter, and only in a few samples from the ore processing plant (Figure 6B). The summer communities were strongly influenced by T, phosphate and Cu concentrations, while the winter communities were influenced by Fe. High pH and high Si concentrations affected the archaeal communities detected in the ore processing plant water samples.

The eukaryotic communities of the ore processing plant separated into a summer cluster influenced most strongly by Cu, and into two winter clusters somewhat influenced by the concentration of thiosulphate (Figure 6C). In the winter, PW and NiTO samples clustered together, whereas CuTa and CuTO samples formed their own group. The summer CuTaF and STaF communities generally affiliated with the other summer samples, in concurrence with the bacterial communities, whereas the CuTaF and STaF winter communities tended to locate closer to other winter communities. The summer and winter communities of the RW and WT fell close to the middle of the plot.

The metagenomic profiles followed the same seasonal trend as seen with the amplicon‐based profiles (Figure 6D). The RW samples were very similar, regardless of season, and fell into the upper right corner of the graph, most strongly influenced by Fe. All the other summer samples but the CuTO were collected to the bottom middle of the graph, influenced most strongly by Cu, K, Sr, Si, S, sulphate and salinity (EC and TDS). In contrast, the winter samples clustered in the upper left corner of the graph, where no significant physicochemical parameter influenced the communities.

### Metagenomic analyses

**FIGURE 7:**
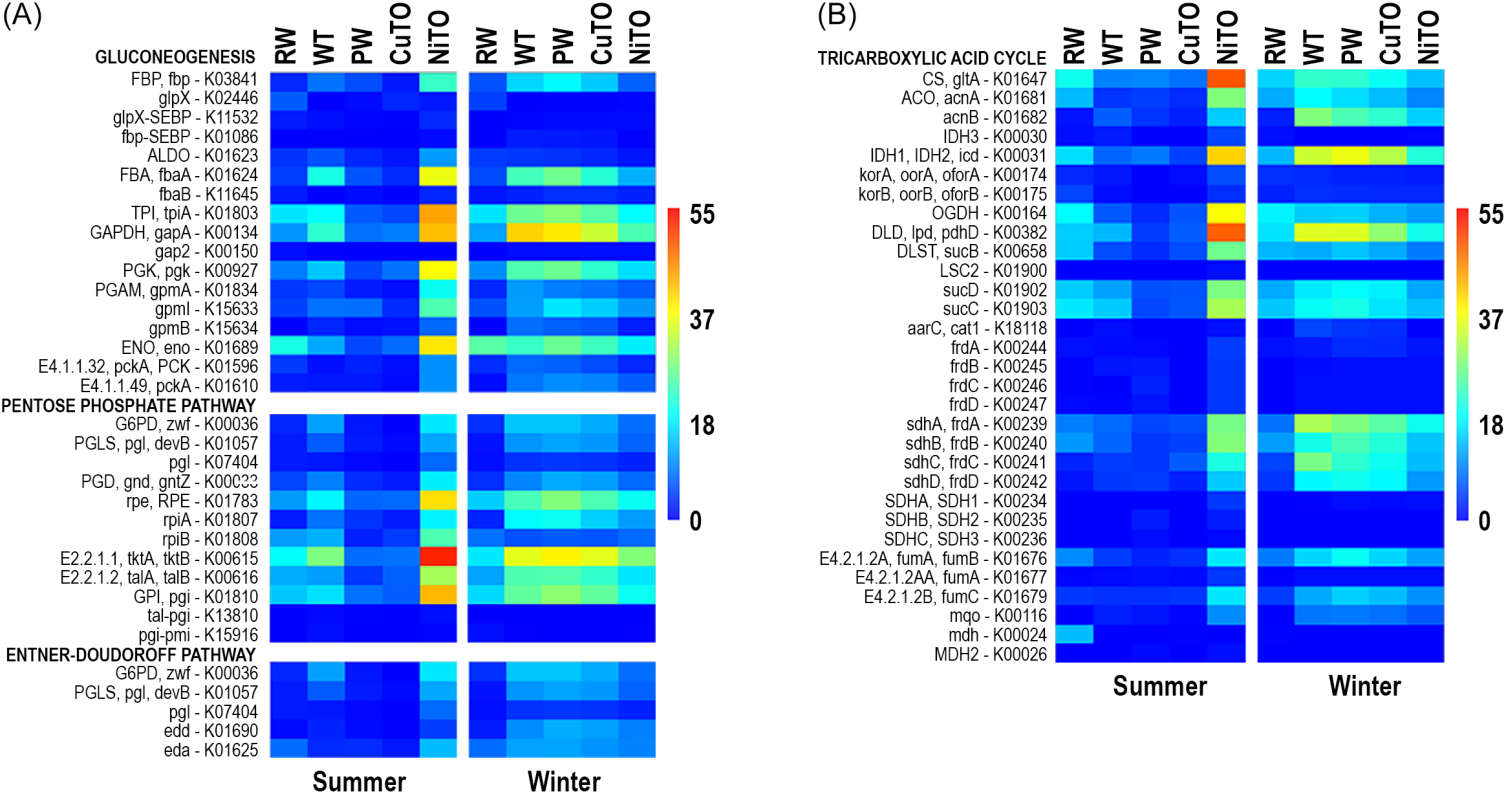
Heatmaps presenting the average detection frequencies of genes involved in (A) gluconeogenesis, pentose phosphate pathway and Entner–Doudoroff Pathway, and (B) tricarboxylic acid cycle (TCA). The colour scale present average detection frequency of genes, that is, the average depth of mapped sequence reads that cover the whole annotated gene. Blue indicates no coverage and red indicates highest average coverage.

The total number of contigs of >2000 bp in length was 35,604, with a N50, N75 and N90 of 8210 bp, 4260 and 3029, respectively, and a total length of the assembly was 238 M bp (Table SA6). The number of contigs >5 kb, >10 kb, >20 kb, >50 kb and <100 kb was 13,337, 4849, 1470, 208 and 51, respectively. The longest contig was 1.16 Mb long. The number of genes predicted by Prodigal in the whole assembly was 251,491. The number of L50, L75 and L90 contigs was 6481; 16,883 and 26,922, respectively. The detection frequencies identified from the metagenomic data showed that the central metabolism pathways. that is, glycolysis, gluconeogenesis, pentose phosphate pathway, Entner–Doudoroff pathway, tricarboxylic acid cycle (TCA), were present in all samples (Figure 7).

**FIGURE 8:**
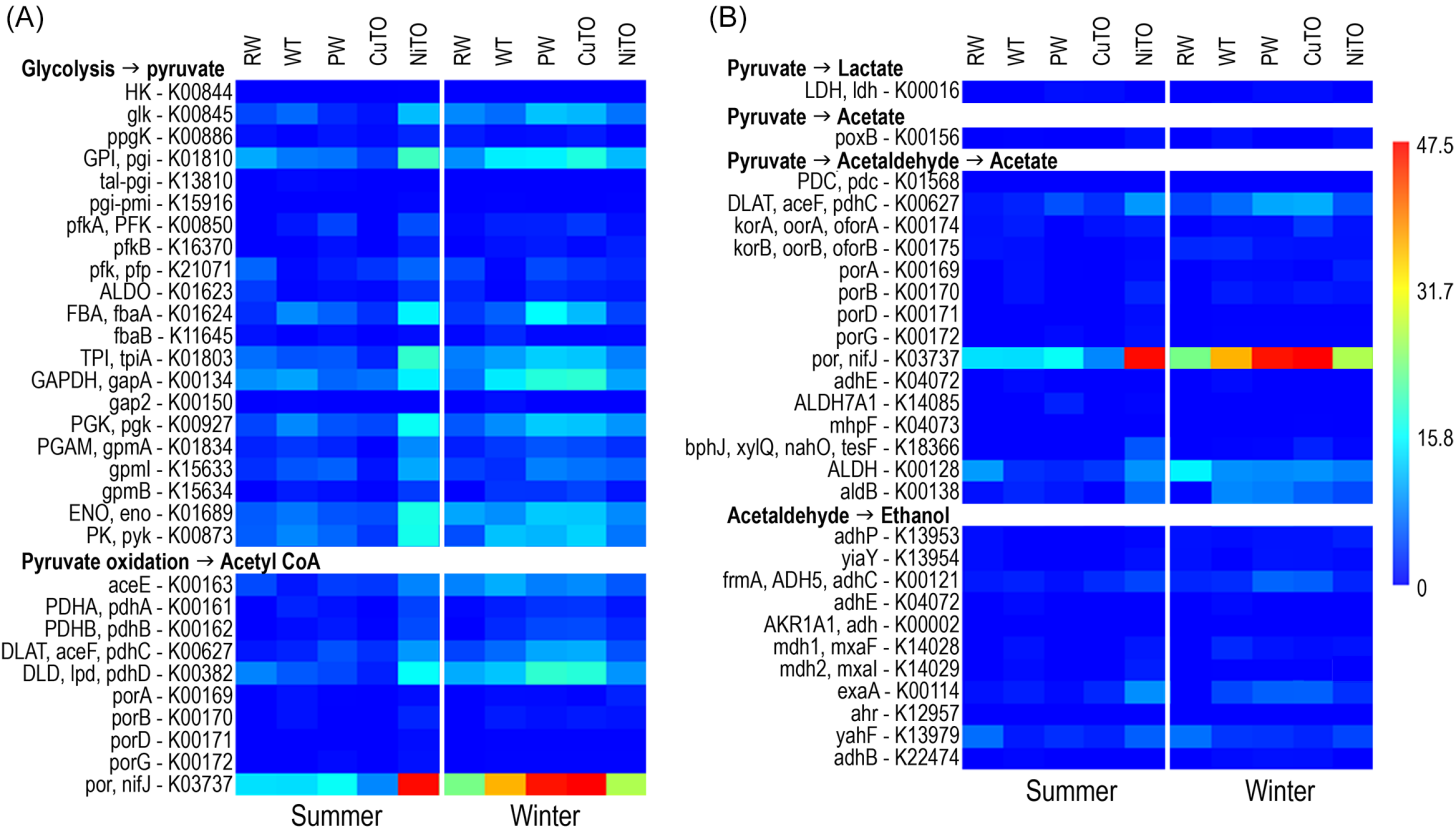
Heatmaps presenting the average detection frequencies of genes involved in (A) glycolysis (conversion of glucose to pyruvate) and pyruvate oxidation to acetyl CoA, and (B) fermentation of pyruvate to lactate, acetate and acetaldehyde and acetaldehyde to acetate or ethanol. The colour scale presents average detection frequency of genes, that is, the average depth of mapped sequence reads that cover the whole annotated gene. Blue indicates no coverage and red indicates highest average coverage.

The pyruvate‐ferredoxin/flavodoxin oxidoreductase, por/nifJ K03737 (Figure 8) that oxidizes pyruvate to acetyl‐CoA was very prominent especially in the winter and in the NiTO summer sample. Gluconeogenesis and pentose phosphate pathway (PPP) were more prominently present in the samples than the glycolysis, again also more frequent in winter. Genes involved in fermentation were less common than genes involved in aerobic sugar metabolisms, indicating that fermentation (e.g., acetate, lactate and ethanol) products would not be produced.

**FIGURE 9:**
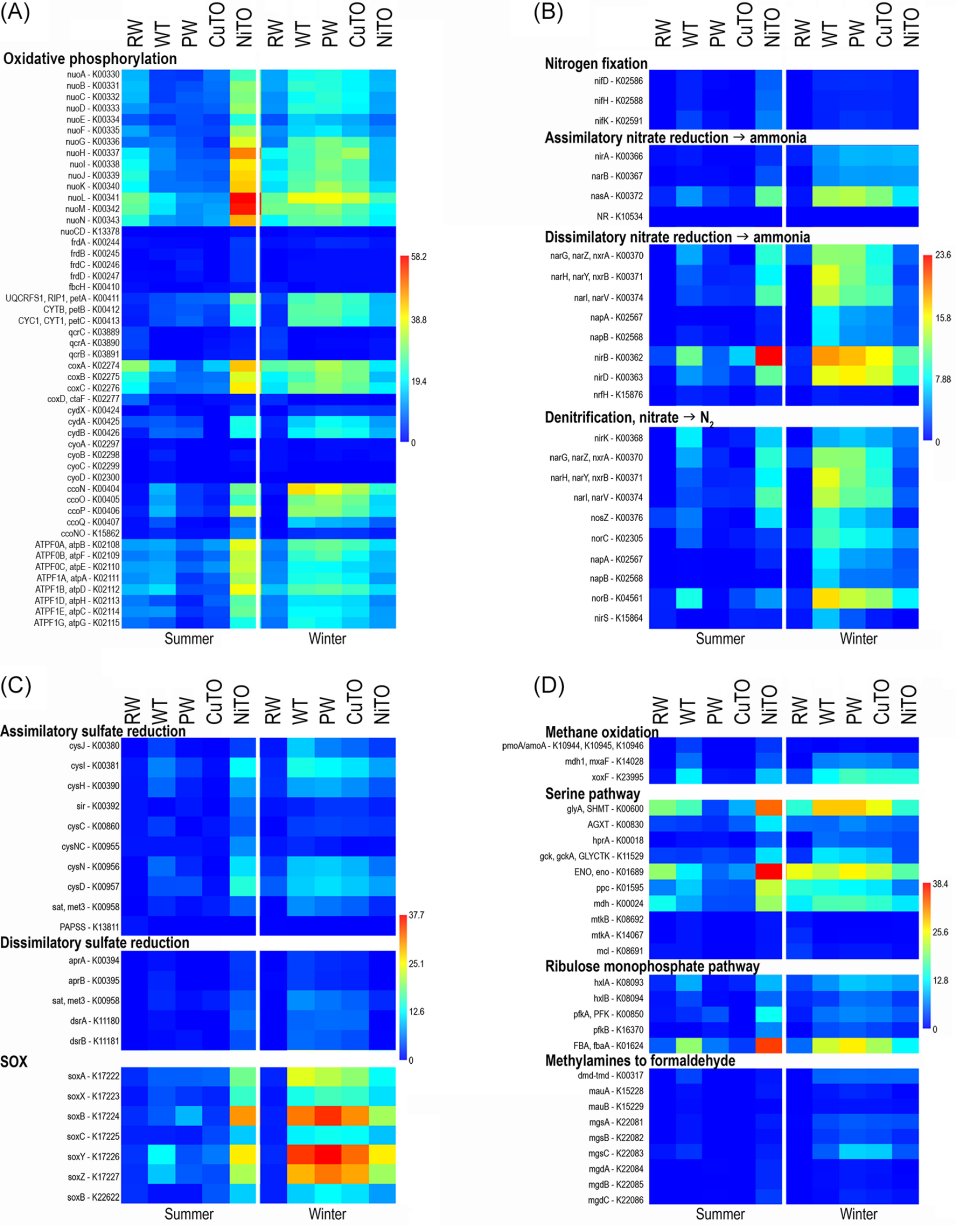
Heatmaps presenting the average detection frequencies of genes involved in (A) oxidative phosphorylation, (B) nitrogen cycling, (C) sulphur cycling and (D) methane cycling. The colour scale presents average detection frequency of genes, that is, the average depth of mapped sequence reads that cover the whole annotated gene. Blue indicates no coverage and red indicates highest average coverage.

Oxidative phosphorylation was the most prominent ATP producing system identified in all samples (Figure 9A). The NADH:quinone oxidoreductase complex was the most common and identified from all samples. The cytochrome c oxidase and F‐type ATPase complexes were also common in all samples, whereas the cytochrome bc1 and cytochrome ud ubiquinol oxidase, the cytochrome c oxidase cbb‐3 type complex were more pronounced in the winter samples and the NiTO summer sample than in other summer samples.

Genes involved in dissimilatory nitrate reduction (DNRA) to ammonia and denitrification were slightly more common in the winter compared to summer samples, but the nitrite reductase genes *nir*B and *nir*D were especially so in the winter WT, PW, CuTO and NiTO samples (Figure 9B). Nevertheless, the summer NiTO had the highest detection rate of the *nir*B. Assimilatory nitrate reduction genes were more prominent in the winter samples compared to the summer samples, with the exception of the RW, from which the detection was low both in the summer and winter. The frequency of genes for nitrogen fixation to ammonia was low in all samples. Genes for assimilatory sulphate reduction were more common than those involved in dissimilatory sulphate reduction (Figure 9C). Nevertheless, the detection frequency of both assimilatory and dissimilatory sulphate reduction genes was higher in winter samples compared to summer samples and more pronounced in the WT, PW and CuTO samples. The summer NiTO sample was again an exception showing the highest detection frequency of both sulphate reduction pathways. Thiosulphate oxidation through the SOX pathway was the most commonly detected sulphur cycling pathway and was detected in all process samples, but not in the RW. The highest frequency of the SOX pathway genes was detected in the winter samples and in the summer NiTO.

Methanogenesis and methane oxidation genes were not detected in the studied samples, but other genes involved in methylotrophy were found (Figure 9D). Methanol dehydrogenase genes (mdh1, *mxa*F, *xox*F) for oxidation of methanol to formaldehyde were detected in the WT and NiTo both in the summer and winter samples and in PW and CuTO in the winter. A small number of *xox*F genes were also detected from the winter RW sample. Formaldehyde was further assimilated via the serine pathway or the RuMP pathway, of which the serine pathway was more prominent. The formaldehyde was oxidized through several steps to L‐malate, which was further most likely fed into the TCA cycle to produce acetyl‐CoA, as the last genes of the serine pathway were only sparsely present. The RuMP pathway was less pronounced than the serine pathway but was nevertheless more frequent in the winter samples than the summer samples, with the exception of the NiTO sample, where the situation was the opposite. The fructose‐bisphosphate aldolase (ALDO) showed a higher frequency than the rest of the RuMP pathway genes. It should also be noted that, for example, the *gly*A, SHMT, ENO and ALDO are also involved in other metabolic pathways, such as the glycolysis, which may overestimate the abundance of certain processes.

**FIGURE 10:**
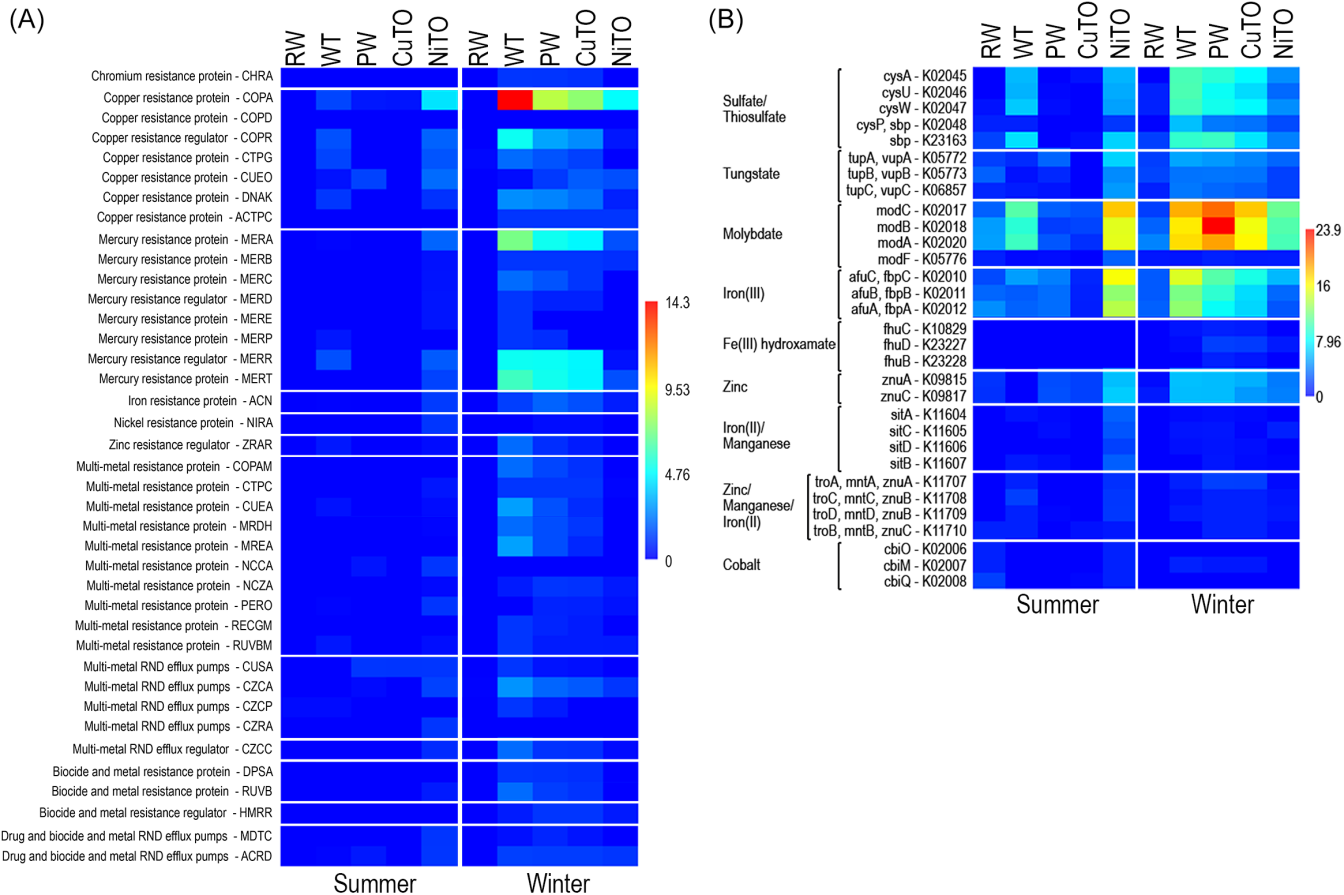
Heatmaps presenting the average detection frequencies of (A) metal resistance genes, and (B) ABC transporters moving metals. The colour scale presents average detection frequency of genes, that is, the average depth of mapped sequence reads that cover the whole annotated gene. Blue indicates no coverage and red indicates highest average coverage.

Metal resistance genes were not very commonly detected in the metagenomes (Figure 10A). However, there was a higher frequency of metal resistance genes in the winter compared to the summer and only in the samples from the Kevitsa process, not in the RW. The most commonly detected metal resistance genes were against Cu and Hg, ABC‐transporters (ATP‐binding cassette transporters), that is, proteins that shuffle compounds through the plasma membrane utilizing ATP as energy, were commonly detected, especially from the winter samples (Figure 10B). Molybdate transporting ABC‐transporters were the most commonly identified ones and were most frequent in the WT, PW, CuTO winter and NiTO summer samples. In addition, summer WT and winter NiTO also contained Mo transporting ABC‐transporter genes, but at a lower frequency, ABC‐transporter genes for Fe(III) were detected at highest frequency from winter WT, PW, CuTO and summer NiTO, but less from the other metagenomes. ABC‐transporter genes for zinc and tungstate were found at low frequency from winter WT, PW, CuTO and summer NiTO and at very low frequency from the other metagenomes. Sulphate and thiosulphate ABC‐transporter genes were detected at highest frequency from summer WT and NiTO and winter WT, PW, CuTO and at a lower frequency from winter NiTO. In the RW samples and summer PW and CuTO, sulphate and thiosulphate ABC‐transporter genes were detected at only very low levels.

In general, the MANOVA analysis of the central metabolism pathways, energy metabolism and metal resistance and transport pathways showed that the highest detection frequency of the glycolysis pathway coincided with the highest detection of the other pathways of the central metabolism. that is, the gluconeogenesis, TCA, PPP, as well as with the oxidative phosphorylation, Fe(III) transport, DNRA, denitrification and SOX (Table 2). Metal resistance correlated strongly with oxidative phosphorylation and SOX, and methane and methanol oxidation (especially the serine pathway) correlated with NADH:quinone oxidoreductase and SOX.

**TABLE 2:** Positive correlation of the metabolic pathways presented in Figures 7, 8, 9, 10. Cells with stars (*) indicate that a statistically significant correlation was found. The number of stars indicates the *p*‐value. *p < 0.05. **p < 0.001. ***p < 0.0001 after Bonferroni correction. Empty cells indicate that no significant correlation was found between the groups. Only pathways with significant correlations with other pathways are shown.

	Glycolysis	TCA	Pentose phosphate pathway	Gluconeogenesis	Metal resistance	Iron(III) transport	Methane and methanol oxidation	DNRA	Denitrification	NADH:Quinone oxidoreductase	Cytochrome c oxidase	Cytochrome c oxidase. cbb3‐type	F‐type ATPase	SOX
Glycolysis		***	***	*		*		*	***	***	***	***	***	***
TCA	***				**		*		*	**	*		*	*
Pentose phosphate pathway	***				**					*				
Gluconeogenesis	*									*				
Metal resistance		**	**							***	**	*	***	**
Iron(III) transport	*													
Methane and methanol oxidation		*								***	*		*	**
DNRA	*													
Denitrification	***	*								*				
NADH:quinone oxidoreductase	***	***	*	*	***		***		*					
Cytochrome c oxidase	***	*			***		*							
Cytochrome c oxidase. cbb3‐type	***				*									
F‐type ATPase	***	*			***		*							
SOX	***	*			***		**							

## DISCUSSION

The mining industry uses large quantities of water in different mining processes, of which the mine operations and concentrations use the major part. In northern regions, water availability is not restricted, but in water scarce areas PW recycling may be a crucial component for operations to proceed. Mines in Finland use 1.2–4.3 m^3^ water per tonne ore (Wessman et al., 2014). The Boliden Kevitsa mine uses approximately 26.4 M m^3^ water per year and recycles up to 97% of the water, taking in only approximately 3.0–3.6% natural raw water (https://www.kaivosvastuu.fi/en/yrityskortti/boliden‐kevitsa‐mining‐oy/#:~:text=Water%20consumption%3A%2026.4%20million%20m3.and%201%20in%20Class%203).

In the Boliden Kevitsa mine's mineral processing plant water loop, a variation in the composition of microbial communities in the summer and winter was clearly seen (Figures 3, 4, 5), although no statistically significant differences were shown for the microbial numbers or alphadiversity metrices. However, in some sample types (e.g., PW, NiTO) some taxa were more prominent in the winter than in the summer. Temperature was the environmental factor that most clearly affected the bacterial community compositions in the summer samples of the Boliden Kevitsa mine's process plant water loop (Figures 3, 4, 5), whereas TC and As influenced the winter communities. The eukaryotic summer communities were also strongly influenced by temperature, but even more affected by Cu. Sulphate, sulphur, TDS and Cu also determined the metagenomic profiles of the summer process samples, with the exception of CuTO, whereas the winter communities appeared not to be as strongly influenced. In all PCoA analyses, iron influenced only the RW bacterial communities and metagenomic profiles, independent of season. In conclusion, it may thus be assumed that temperature and Cu concentration in the PW may exhibit the highest pressure of the studied parameters on the major microbial communities in this environment, where nutrients, such as phosphorus were scarce. Furthermore, the results clearly show that the raw water that the Boliden Kevitsa mine plant uses has low effect on the processing plant microbiome and it has very different bacterial, archaeal and eukaryotic composition (Figures 3, 4, 5, 6), community metabolism (Figures 7, 8, 9, 10) as well as physicochemical characteristics (Table 2) compared to any of the plant process samples either in summer or winter time.

In a previous study spanning over only 2 months (August–September, 2017), the microbial community was not notably different between the sampling times in PW, NiTO and STa in Boliden Kevitsa mine (Bomberg et al., 2020). The summer sampling campaign of the present study (28 August 2018) coincides with the same season as in the previous study and the microbial communities in the PW samples were similar in general, although the older samples had a clear dominance of Rhodobacteraceae bacteria (up to 36 and 42% in the PW and Ni thickener overflow, respectively) whereas in the 2018 summer samples, the relative amount of Rhodobacteraceae was 14.6 and 11.5% in the PW and NiTO, respectively, and 6.5 and 9.6%, respectively, in the winter samples (Table S1, Figure S1). In contrast, bacteria belonging to the Xanthobacteraceae were prominent in the 2018 summer PW and NiTO samples (up to 16.1 and 26.4%, respectively), whereas this group was represented by at most 9.0 and 5.3% in PW and NiTO in 2017 and were almost absent in the winter samples of 2018. Rhodobacteraceae is a large bacterial family containing a great number of species that are involved in sulphur cycling in aquatic environments (Pujalte et al., 2014). Members of the Xanthobacteraceae oxidize reduced sulphur compounds, are methylotrophic and chemoheterotrophic or chemolithoautotrophic (Oren, 2014). These were also present in the other sample types. Generally, these microbial groups have prevailed in the processing plant ecosystem over a whole year, although their relative abundances have changed. Thus, they seem to be an enduring part of the microbial community regardless of or more likely because of the process and environmental parameters such as temperature and metal concentrations which they have adapted to.

Methylotrophic bacteria, such as species of the Methylophiliaceae, and thiosulphate oxidizing bacteria, such as species of the Sulfurimonadaceae, were common and found together in the plant and process samples (Supplement B). This was also reflected in the metagenomes, where methanotrophy/methylotrophy correlated significantly with the SOX pathway (*p* < 0.001). The Methylophiliaceae also reduce nitrate (Kalyuhznaya et al., 2009). Nitrate reduction to ammonia and denitrification, although not with statistical significance, were common processes detected, especially in the winter, when, for example, the Methylophiliaceae were common (Figure SA3, Supplement B). The Boliden Kevitsa mine PWs contained high amounts of organic carbon, thiosalts and nitrate‐N, which likely cause the high prevalence of these metabolic pathways. Chen et al. (Chen et al., 2008) showed that microbial consortia may remove organic carbon, nitrogen and sulphur compounds simultaneously, where nitrogen and carbon leave the system as N_2_ and CO_2_, respectively, and sulphur is precipitated as S^0^. Many sulphur oxidizers, such as *Thiobacillus denitrificans*, *Sulfuricurvum* sp. and *Sulfurimonas denitrificans* may oxidize sulphide with nitrate as electron acceptor (Handley et al., 2014; Sievert et al., 2008; Wang et al., 2005), and members of these genera were also detected at high relative abundance in Boliden Kevitsa mine. The reason for the pronounced presence of methylotrophic bacteria is not clear. However, CMC is used as collector in the Boliden Kevitsa mine flotation process and part of the added CMC remains in the water circuit. Cellulosic material can be degraded to different smaller polymers and alcohols, such as methanol by cellulolytic microorganisms (Khoo et al., 2016). Genes for cellulolytic enzymes were present especially in the winter samples. Reduced sulphur compounds used by the sulphur oxidizing bacteria are most likely originating from the processed ore, whereas the nitrogen compounds are remnants from blasting chemicals. Both the 16S rRNA gene sequence data and the metagenomic sequence data indicate that methylotrophy, sulphur oxidation and nitrate reduction were important metabolic strategies both in the summer and winter communities. This was also supported by the metabolic predictions on the bacterial communities detected in 2017 (Bomberg et al., 2020). The metagenomic data indicate a stronger presence of these pathways in the winter, although this may be a technical issue due to the need to amplify the DNA samples for the metagenomic analyses. Nevertheless, the microbial community analyses suggest that different microbial groups are responsible for these metabolic functions depending on the season, which is influenced by factors such as temperature, precipitation and primary production rates.

The microbial communities had a high aptitude for iron(III) and molybdate transport as well as a high level of copper (*cop*A, *cop*D) and mercury (*mer*A, *mer*R and *mer*T) resistance, especially in the winter communities. Interestingly, the Cu content of the winter samples was generally below the limit of detection (<3.0 μg L^−1^), whereas in the summer the concentration was between 7.7 and 15 μg L^−1^. Iron and molybdate are essential nutrients in all life and are needed in many enzymes in the major metabolic processes in the cell (Andrews et al., 2003; Schwarz et al., 2009). The concentration of iron in the PW and plant water was generally below the detection limit of the assay, rendering the ability to scavenge iron from the environment important. Siderophores are also important metal harvesting peptides that chelate essential metal ions which can then be imported into the cell (Schalk et al., 2011). Siderophores are very diverse which makes their identification from metagenomes challenging and they were not identified in the metagenomes from Boliden Kevitsa mine. Copper is also present in different enzymes in many microorganisms and may be present at high concentrations in the cell. However, too high concentration is toxic for microorganisms and must be removed by, for example, efflux pumps, such as the CopA ATPase. In Boliden Kevitsa mine, *cop*A was more pronounced in the winter than in the summer.

No great differences in the enrichment of genes involved in central energy metabolism were seen, except that there appeared to be a slightly higher overall enrichment in these genes in the winter samples compared to the summer samples. However, the *por*A, *nif*J gene (K03737) oxidizing pyruvate to acetyl‐CoA was noticeably prominent, especially in winter. Acetyl‐CoA is an important acetyl‐group bearing molecule that participates in numerous metabolic processes in the microbial cells and serves as raw material for the generation of carbohydrates, proteins and fatty acids but can also be used for the production of acetate.

ATP was produced with the NADH:quinone oxidareductase, cytochrome c oxidase, CSB3‐type cytochrome oxidase and F‐type ATPase with some variation between the samples. Interestingly, the genes involved in glycolysis and TCA, from which the electrons for ATP synthesis via oxidative phosphorylation are sourced were not as common.

DNRA and denitrification were common, especially in the process winter samples and the summer NiTO, coinciding with the highest levels of genes of the NADH:quinone oxidareductase, cytochrome c oxidase, CSB3‐type cytochrome oxidase and F‐type ATPase.

Archaea were found mainly in the RW sample, both in summer and winter. Most of the archaeal sequences belonged to the nano‐sized, heterotrophic and most likely synergistic phylum of Woesearchaea, which are ubiquitously found in aquatic environments (Liu et al., 2017). Seasonality was observed in the structure and presence of taxa in the archaeal communities.

The amount of Eukaryota in the samples was low and did not change with season. The majority of the obtained eukaryotic sequences, in some cases up to 98%, could not be assigned to any known taxa, except unclassified fungi, but these were clearly affected by the concentration of Cu, especially in the summer. Fungi often secrete organic acids as a by‐product of their metabolisms or, for example, as a means for solubilizing minerals and chelate metal ions (Fomina et al., 2005; Gadd, 2007). Fungi rapidly react to environmental change, such as organic matter input (e.g., increased pollen input in early summer) and have important roles in the food webs and organic matter recycling (Grossart et al., 2019). The effects of fungal and eukaryotic activity and metabolism in minerals processing are poorly understood but they are constantly found in mining and mineral processing environments when looked for (e.g., Ehrman et al., 2008; Miettinen et al., 2021; Mohammadian et al., 2017). Fungi may, for example, precipitate minerals (Ehrman et al., 2008) and thus cause clogging, or produce organic acids (Arwidsson et al., 2010), which may chelate metals or function as carbon source for bacteria and archaea. In suitable conditions, they can affect both water and mineral environments, even at low abundance.

A new DNA extraction protocol using phenol and anionic nanocellulose was used to extract the DNA from the CuTa and STa samples, which had the highest proportion of mineral solids (Bomberg & Miettinen, 2023). Slurries of the samples were used and the DNA extraction results were compared to those obtained with the commercial DNA extraction kit used for the filtered samples. The amount of DNA obtained was low with the phenol extraction, but still higher than with the commercial kit. The DNA extracts from the commercial kits of these two samples produced amplicon sequence reads only from a low number of parallel samples, whereas the majority of the parallels treated with the phenol extraction protocol resulted in adequate number of sequence reads in the case of bacteria and eukaryotes. In addition, the number of bacterial, archaeal and eukaryotic marker gene copies were below the limit of detection in the qPCR assay when run with the commercial DNA kit extracts of the winter samples, whereas bacteria and eukaryotes were detected from the phenol‐extracted samples. *Thiobacillus* appeared to be especially well detected in the CuTaF samples but were much less common in the other sample types and were not detected from the CuTa. However, overall, the microbial communities in the different sample types were very similar; the microbial communities detected using the phenol‐protocol included. This suggests that the microorganisms present in the water also attach readily to mineral particles. Due to the long procedure of the customized DNA extraction protocol and the use of nanocellulose, negative reagent controls were considered highly important. These negative reagent controls were also submitted to qPCR and amplicon sequencing analysis and background from the controls was removed from the samples. The protocol for detaching microbial cells or extracting DNA from mineral samples may be challenging, as both cells and DNA readily adsorb to mineral surfaces (Bomberg & Miettinen, 2023; Le et al., 2020). This needs to be considered when examining the microbial population of mineral bearing samples.

## CONCLUSIONS

The microbial communities in the Boliden Kevitsa mine's minerals processing water loop are highly diverse with cell numbers typical for oligotrophic freshwater environments. There was no great difference in the microbial community sizes between summer and winter, but the community structures were clearly different between the seasons. Methylotrophy, sulphur oxidation and nitrate reduction were prominent metabolic traits in the water samples, with a more distinct detection in the metagenomic data of the winter samples. However, the taxonomical 16S rRNA gene data of the microbiomes indicated that methylotrophic, sulphur oxidizing and nitrate reducing bacteria dominated both in the summer and in the winter, but the species that performed these reactions were different depending on the season. The greater part of the obtained eukaryotic sequences could only be assigned to unclassified fungi, but they were clearly affected by the concentration of Cu, especially in the summer. Archaea were present but did not have a role in the minerals processing environment. The plant raw water source, that is, RW, had different microbiome from the process samples, and it can be concluded that microbiomes in the Boliden Kevitsa mine's minerals processing water loop have metabolic capacities which develop within the process itself over time in response to the conditions in the processing plant, water chemistry, used chemicals, ore properties and seasonal variation.

## AUTHOR CONTRIBUTIONS

**Malin Bomberg:** Conceptualization (equal); formal analysis (lead); investigation (lead); methodology (lead); validation (equal); visualization (lead); writing – original draft (lead); writing – review and editing (equal). **Hanna Miettinen:** Conceptualization (equal); data curation (equal); investigation (equal); methodology (equal); validation (equal); writing – review and editing (equal). **Päivi Kinnunen:** Conceptualization (equal); funding acquisition (lead); investigation (equal); project administration (lead); validation (equal); writing – review and editing (equal).

## CONFLICT OF INTEREST STATEMENT

The authors declare no conflicts of interest.

## Supporting information


**DATA S1:** Supporting Information.

**DATA S2:** Supporting Information.

**DATA S3:** Supporting Information.

## Data Availability

All supporting data, code, and protocols are provided within the article and supplementary data file. All sequence data have been deposited in the European Nucleotide Archive under accession number PRJEB61213: https://www.ebi.ac.uk/ena/browser/view/PRJEB61213.
