# Supplementary material for: Seasonal variation in metabolic profiles and microbial communities in a subarctic ore processing plant
Source: Environ Microbiol Rep. 2024 Jun 23;16(3):e13284. doi: 10.1111/1758-2229.13284 (PMC11194043; doi:10.1111/1758-2229.13284)
Supplement: Supplementary file 1 — DATA S1: Supporting Information. [file EMI4-16-e13284-s003.docx]

**Supplementary A.**

**Table SA1.** The types of samples and sample volumes and DNA extraction protocols used.

| Sample name | Sampling spots | Summer  sample volume and type | Winter  sample volume and type | DNA extraction method |
| --- | --- | --- | --- | --- |
| RW | River water  (Raw water) | 3 x 400 mL water on Sterivex filters | 3 x 500 mL water on Sterivex filters | NucleoSpin Soil DNA extraction kit |
| WT | Water tank | 3 x 500 mL water on Sterivex filters | 3 x 250 mL water on Sterivex filters | NucleoSpin Soil DNA extraction kit |
| PW | Process water | 3 x 500 mL water on Sterivex filters | 3 x 500 mL water on Sterivex filters | NucleoSpin Soil DNA extraction kit |
| CuTa | Cu flotation tailings | 3 x 5 mL slurry. centrifuged. pellet for DNA extraction | 3 x 5 mL slurry. centrifuged. pellet for DNA extraction | NucleoSpin Soil DNA extraction kit |
|  |  | 3 x 32.5 mL slurry | 3 x 32.5 mL slurry | Customized DNA extraction protocol (Bomberg and Miettinen, 2023) |
| STa | Sulfur flotation tailings | 3 x 5 mL slurry centrifuged. pellet for DNA extraction | 3 x 5 mL slurry centrifuged. pellet for DNA extraction | NucleoSpin Soil DNA extraction kit |
|  |  | 3 x 27 mL slurry | 3 x 32.5 mL slurry | Customized DNA extraction protocol (Bomberg and Miettinen, 2023) |
| CuTO | Cu thickener overflow | 3 x 250 mL water on Sterivex filters | 3 x 250 mL water on Sterivex filters | NucleoSpin Soil DNA extraction kit |
| NiTO | Ni thickener overflow | 3 x 250 mL water on Sterivex filters | 3 x 250 mL water on Sterivex filters | NucleoSpin Soil DNA extraction kit |

**Table SA2.** The concentration of DNA obtained from the samples. All samples were eluted in 100 µL elution buffer. The concentrations are presented as ng/µL and sample size in mL.

|  | Summer | | | |  | Winter | | | |
| --- | --- | --- | --- | --- | --- | --- | --- | --- | --- |
|  | Sample size, mL | A | B | C |  | Sample size, mL | A | B | C |
| RW | 400 | 3.43 | 3.05 | 3.45 |  | 500 | 6.2 | 5.5 | 5.1 |
| WT | 500 | 2.73 | 2.37 | 3.0 |  | 250 | 4.5 | 4.9 | 6.7 |
| PW | 500 | 0.17 | 0.132 | 0.06 |  | 500 | 6.6 | 4.5 | 4.0 |
| CuTa | 5 | bd | bd | bd |  | 5 | 0.232 | 0.230 | bd |
| CuTaF | 32.5 | 0.228 | 0.227 | 0.212 |  | 37.5 | 0.232 | 0.230 | 0.194 |
| STa | 5 | bd | bd | bd |  | 5 | 0.194 | 0.233 | bd |
| STaF | 32.5 | 0.230 | 0.189 | 0.154 |  | 32.5 | 0.194 | 0.233 | 0.184 |
| CuTo | 250 | bd | bd | bd |  | 250 | bd | bd | bd |
| NiTo | 250 | 2.28 | 1.6 | 1.4 |  | 250 | 2.2 | 2.8 | 4.1 |

**Table SA3.** The number of bacterial 16S rRNA gene sequence reads (Sequences), observed number of OTUs (OTUs), estimated number of OTUs (Chao1, ACE) and Shannon’s diversity index (Shannon) for the bacterial communities. Only samples with >200 sequence reads were included in the analyses. Samples from which only one or two of the replicates provided sequence data are indicated with * and **, respectively.

|  | **Sequences** | **OTUs** | **Chao1** | **ACE** | **Shannon** |
| --- | --- | --- | --- | --- | --- |
| **Summer** |  |  |  |  |  |
| RW | 3220 -14586 | 434 - 869 | 631 - 1159 | 1225 | 4.2 - 4.3 |
| WT | 2601 - 8298 | 463 - 868 | 704 - 1142 | 700 - 1202 | 4.3 - 4.6 |
| PW | 2455 - 7330 | 299 - 544 | 445 - 805 | 487 - 870 | 3.9 - 4.1 |
| CuTa | 17 - 41** |  |  |  |  |
| CuTaF | 1194 - 2266 | 192 - 233 | 262 - 328 | 271 - 341 | 3.8 - 3.9 |
| STa | 16 - 48 |  |  |  |  |
| STaF | 2229 - 3466 | 185 - 302 | 234 - 487 | 260 - 496 | 3.1 - 4.2 |
| CuTO | 2211 - 3032 | 175 - 218 | 247 - 382 | 252 - 355 | 2.7 - 2.9 |
| NiTO | 10618 - 21601 | 760 - 1137 | 1168 - 1479 | 1646 - 1292 | 3.9 - 4.2 |
| **Winter** |  |  |  |  |  |
| RW | 7474 - 15958 | 660 - 815 | 897 - 1057 | 960 - 1148 | 4.5 - 4.7 |
| WT | 2035 - 6009 | 95 - 200 | 150 - 305 | 183 - 353 | 1.8 - 2.8 |
| PW | 3614 - 10178 | 176 - 326 | 270 - 469 | 315 - 558 | 2.6 - 2.9 |
| CuTa | 22 - 208 | 25* | 31* | 44* | 2.3 |
| CuTaF | 672 - 1848 | 81 - 184 | 107 - 279 | 124 - 296 | 3.2 - 3.6 |
| STa | 17 - 706 | 53 - 74** | 99 - 109** | 92 - 129** | 2.5 - 3.6** |
| STaF | 121 - 1446 | 79 - 197** | 140 - 308** | 143 - 338** | 3.3 - 3.5** |
| CuTO | 63 - 4494 | 127* | 154* | 160* | 2.5* |
| NiTO | 304 - 4479 | 45 - 185 | 133 - 288 | 105 - 304 | 2.3 - 2.4 |

**Table SA4.** The number of archaeal 16S rRNA gene sequence reads (Sequences), observed number of OTUs (OTUs), estimated number of OTUs (Chao1, ACE) and Shannon’s diversity index (Shannon) for the archaeal communities. Only samples with >200 sequence reads were included in the analyses. Samples from which only one or two of the replicates provided sequence data are indicated with * and **, respectively.

|  | **Sequences** | **OTUs** | **Chao1** | **ACE** | **Shannon** |
| --- | --- | --- | --- | --- | --- |
| **Summer** |  |  |  |  |  |
| RW | 217 - 318 | 72 - 90 | 90 - 115 | 97 - 124 | 3.9 - 4.1 |
| WT | 67 - 100 |  |  |  |  |
| PW | 3 - 17 |  |  |  |  |
| CuTa | 1* |  |  |  |  |
| CuTaF |  |  |  |  |  |
| STa |  |  |  |  |  |
| STaF | 2* |  |  |  |  |
| CuTO |  |  |  |  |  |
| NiTO | 70 - 156 |  |  |  |  |
| **Winter** |  |  |  |  |  |
| RW | 547 - 907 | 167 - 213 | 202 - 239 | 220 - 257 | 4.8 - 5.0 |
| WT | 4 - 207 | 31* | 37* | 42* | 2.6* |
| PW | 184 - 317 | 28 - 32** | 35 - 37** | 40 - 42** | 2.5 - 2.6** |
| CuTa |  |  |  |  |  |
| CuTaF | 2* |  |  |  |  |
| STa | 1* |  |  |  |  |
| STaF | 2* |  |  |  |  |
| CuTO |  |  |  |  |  |
| NiTO | 2 - 4 |  |  |  |  |

**Table SA5.** The number of eukaryotic 5.8S rRNA gene sequence reads (Sequences), observed number of OTUs (OTUs), estimated number of OTUs (Chao1, ACE) and Shannon’s diversity index (Shannon) for the eukaryotic communities. Only samples with >200 sequence reads were included in the analyses. Samples from which only one or two of the replicates provided sequence data are indicated with * and **, respectively.

|  | **Sequences** | **OTUs** | **Chao1** | **ACE** | **Shannon** |
| --- | --- | --- | --- | --- | --- |
| **Summer** |  |  |  |  |  |
| RW | 691 - 778 | 61 - 75 | 71 - 90 | 75 - 103 | 2.5 - 2.7 |
| WT | 2447 - 3547 | 235 - 265 | 303 - 333 | 328 - 331 | 3.6 - 3.9 |
| PW | 1256 - 1937 | 85 - 103 | 123 - 192 | 120 - 203 | 1.5 – 2.0 |
| CuTa | 2 - 127 |  |  |  |  |
| CuTaF | 895 - 2744 | 23 - 58 | 41 - 77 | 60 - 88 | 1.2 - 2.0 |
| STa | 1 - 2** |  |  |  |  |
| STaF | 1363 - 2711 | 18 - 39 | 21 - 50 | 25 - 60 | 0.9 - 2.0 |
| CuTO | 812 - 1266 | 32 - 74 | 42 - 136 | 50 - 136 | 1.4 - 2.5 |
| NiTO | 1640 - 2614 | 133 - 203 | 179 - 251 | 191 - 276 | 2.5 - 2.8 |
| **Winter** |  |  |  |  |  |
| RW | 122 - 223 | 20* | 21* | 22* | 2.2* |
| WT | 347 - 664 | 43 - 54 | 50 - 69 | 53 - 70 | 2.4 - 2.9 |
| PW | 526 - 10579 | 36 - 114 | 47 - 122 | 49 - 124 | 1.8 - 2.6 |
| CuTa | 236 - 904 | 4 - 15 | 4 - 15 | 5 - 15 | 0.08 - 2.0 |
| CuTaF | 20-2803** | 34* | 34* | 35* | 1.4* |
| STa | 1 - 515 | 7* | 7* | 7.6* | 0.7* |
| STaF | 520 - 630** | 10 - 18** | 11 - 23** | 13 - 23** | 0.2 - 1.0** |
| CuTO | 580 - 1527 | 19 - 36 | 23 - 43 | 24 - 45 | 0.9 - 1.3 |
| NiTO | 385 - 1553 | 31 - 84 | 48 - 92 | 40 - 102 | 1.8 - 2.5 |

***Metagenomic analyses***

The quality of the sequence data was initially checked for the paired reads using Fastqc [1]. The paired reads were merged using SeqPrep [2] using default parameters, resulting in a merged.fastq file, and unmerged .fastq files for the single reads files. The merged and unmerged sequence reads were trimmed using Trimmomatic [3] run in single mode and paired end mode for the merged and unmerged data, using average quality scores of 20, cropping 12 nucleotides from the beginning and 3 nucleotides from the end of the reads and using a minimum length of 75 nucleotides.

The data was prepared for further analysis with anvi’o [4] by combining all F reads. R reads and merged reads into combined F read, R read and merged read .fastq files, respectively, for co-assembly. The .fastq data was assembled with the MEGAHIT assembler [5] using default parameters and allowing for a minimum contig length of 1000 nt. Assembly statistics were obtained using MetaQuast [6]. Because the data was to be analysed using anvi’o, the co-assembly contig names were formatted using anvi’o’s anvi-script-reformat-fasta command with the --simplify-names flag and only keeping contigs longer than 2500 nt (-l 2500). The F, R and merged reads were separately mapped to the refined co-assembly for each sample using Bowtie2 [7]. The co-assembly was first indexed using bowtie2-build. The co-assembly index data was subsequently used for all samples. The F, R and merged read data of each sample was thereafter mapped to the co-assembly contigs with the bowtie2 command using --no-unal -D 20 -R 3 -N 1 -L 20 -i S,1,0.50 for a .sam output. The .sam output was converted to .bam using samtools, and was further sorted using the samtools sort command.

The data was further analysed using anvi’o v. 6.1 [4]. A contigs database (CONTIGS.DB) was generated based on the refined co-assembly using anvi’o’s anvi-gen-contigs-database command. Gene calls in the refined co-assembly were identified using the Hidden Markov model (hmm) with anvi-run-hmms after which nucleotide and amino acid sequences were extracted with anvi-get-sequences-for-gene-calls.

The taxonomical composition of the refined co-assembly gene calls was annotated using Kaiju v 1.7.3 using the nr_euk database version 2019-06-25 [8] and Krona [9]. The Kaiju taxonomy for the gene calls were imported into the CONTIGS.DB using anvi-import-taxonomy-for-genes.

Functional annotations were done against the ncbi COGs database (latest version 2003-2014) and Pfams (version 32) [10] using anvi’o. First, the databases were set up using the anvi-setup-ncbi-cogs and anvi-setup pfams commands, respectively. Thereafter the annotations were run with the commands anvi-run-ncbi-cogs and anvi-run-pfams with the CONTIGS.DB as input file. Single copy genes (scg) were identified with the anvi-setup-scg-databases after which the scg in the CONTIGS.DB were identified with the anvi-run-scg-taxonomy. The COGs, Pfams and scg annotations were automatically imported to the CONTIGS.DB. In addition, functional annotations on the nucleotide and amino acid sequences of the gene calls was performed using PROKKA version 1.14.5 [11] on the USEGALAXY.org server and GHOSTKOALA [12] server, respectively. The PROKKA data was incorporated into the CONTIGS.DB by first parsing the .gff file using the gff_parser.py script (courtesy of Antti Karkman <https://raw.githubusercontent.com/karkman/gff_parser/master/gff_parser.py>) and then using the anvi-import-functions script to import the gene annotation data to the CONTIGS.DB. For the GHOSTKOALA analysis, the amino acid sequence identifiers were first modified from numbers to genecall_number after which the amino acid sequence data was uploaded to GhostKoala for annotation towards the genus_prokaryotes + family_eukaryotes + viruses database. The produced user_ko.txt file was parsed using the GhostKoalaParser (<https://github.com/edgraham/GhostKoalaParser.git>). First a ko00001.keg file was obtained (<https://www.genome.jp/kegg-bin/download_htext?htext=ko00001&format=htext&filedir>=), which was parsed with the code (courtesy of Elaina Graham <http://merenlab.org/2018/01/17/importing-ghostkoala-annotations/>);

kegfile="ko00001.keg"

while read -r prefix content

do

case "$prefix" in A) col1="$content";; \

B) col2="$content" ;; \

C) col3="$content";; \

D) echo -e "$col1\t$col2\t$col3\t$content";;

esac

done < <(sed '/^[#!+]/d;s/<[^>]*>//g;s/^./& /' < "$kegfile") > KO_Orthology_ko00001.txt

The generated KO_Orthology_ko00001.txt was converted to anvi’o compatible format using the GhoasKoalaParser phyton script ‘python KEGG-to-anvio --KeggDB KO_Orthology_ko00001.txt

-i user_ko.txt -o KeggAnnotations-AnviImportable.txt. The KeggAnnotations-AnviImportable.txt was used with the anvi-import-functions script to import the gene annotation data to the CONTIGS.DB.

The individually Bowtie2 mapped samples were combined to the CONTIGS.DB to generate an anvi’o profile database (PROFILE.DB) using the anvi-profile command and the sorted.bam files for each sample. Gene coverages and detection in the tested samples was extracted from the anvi’o PROFILE.DB and CONTIGS.DB using anvi-export-gene-coverage-and-detection. The metagenomic data is presented as detection frequencies, which indicate how deeply a specific gene was completely covered by mapping the sequence reads to the contigs.

**Table SA6.** The number of original sequence reads, quality trimmed and merged or unmerged sequence reads and mean coverage of sequence reads to the co-assembly per sample.

|  | Summer | | | | | Winter | | | | | |  |
| --- | --- | --- | --- | --- | --- | --- | --- | --- | --- | --- | --- | --- |
|  | RW | WT | PW | CuTO | NiTO | | RW | WT | PW | CuTO | NiTO | |
| Original number of sequence reads (million reads) | 11.67 | 12.49 | 10.58 | 12.24 | 16.15 | | 10.72 | 14.99 | 14.03 | 11.67 | 10.96 | |
| Number of trimmed and merged sequence pairs (million reads) | 3.24 | 3.45 | 2.45 | 3.19 | 5.25 | | 2.53 | 3.82 | 3.32 | 3.01 | 2.55 | |
| Number of trimmed and unmerged sequence pairs (million reads) | 8.33 | 8.93 | 8.01 | 8.94 | 10.75 | | 8.04 | 11.06 | 10.59 | 8.51 | 8.33 | |
| Mean coverage | 3.13 | 5.18 | 2.62 | 2.97 | 6.72 | | 3.70 | 6.56 | 7.93 | 5.23 | 5.41 | |

**Supplementary figures.**


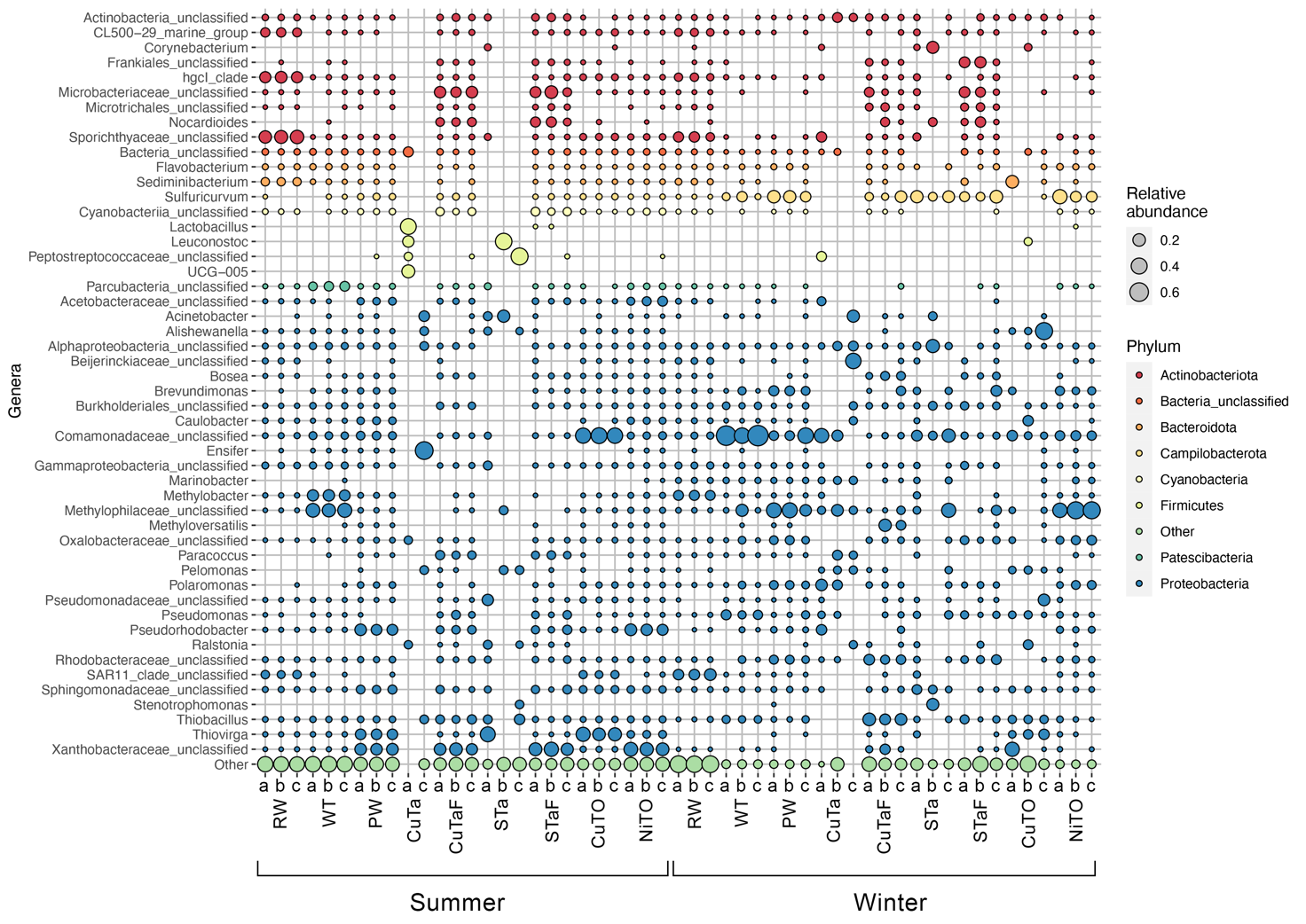


**Figure SA1.** The relative abundance of the 50 most prominent bacterial genera per sample. “Other” indicate all other less abundant genera.


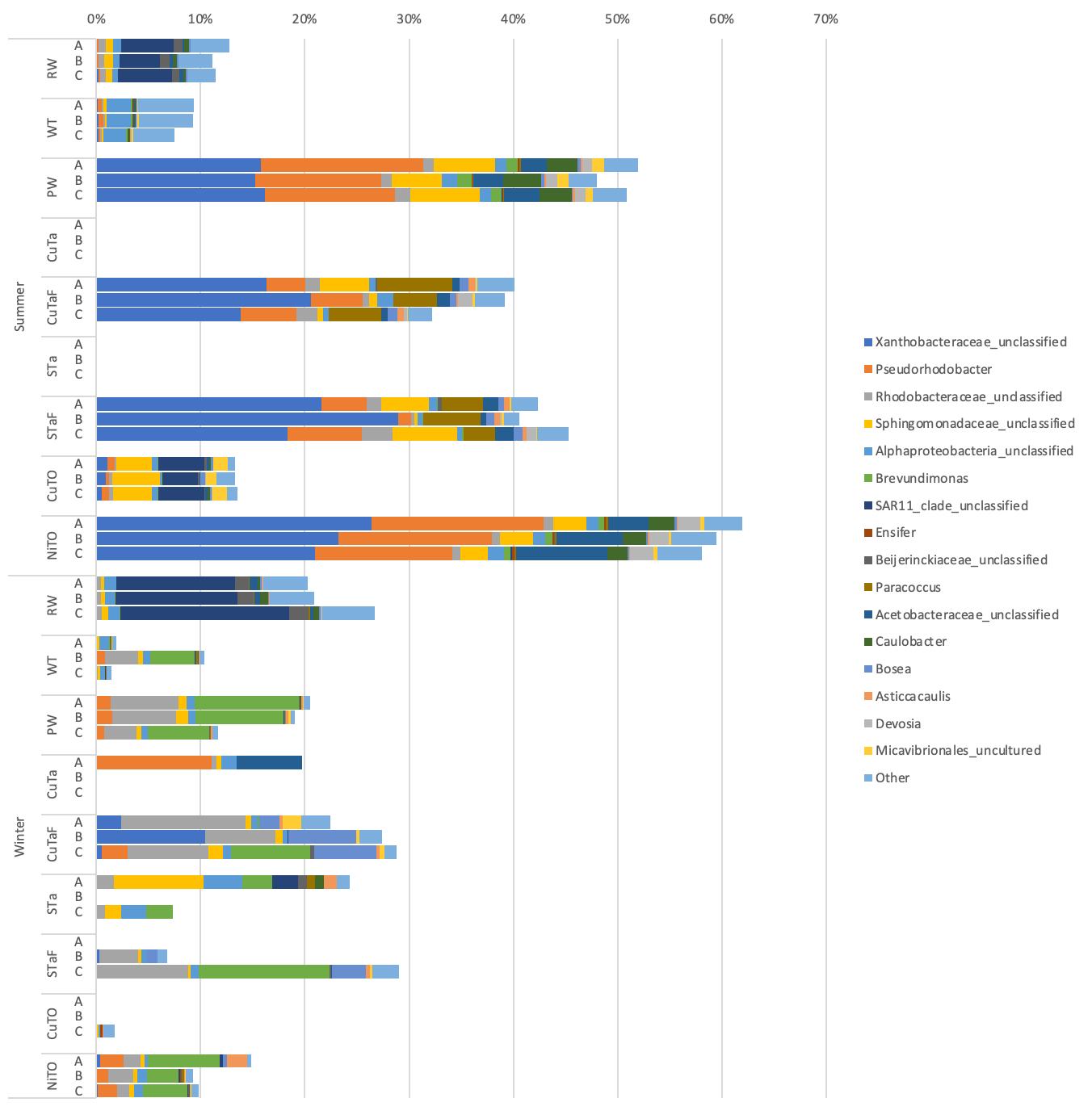


**Figure SA2.** The relative abundances of alphaproteobacterial lineages identified from the summer and winter samples by amplicon sequencing of the bacterial 16S rRNA genes. The ‘Other’ category sums up all alphaproteobacterial taxa representing less than 0.1% of the bacterial community in all samples.


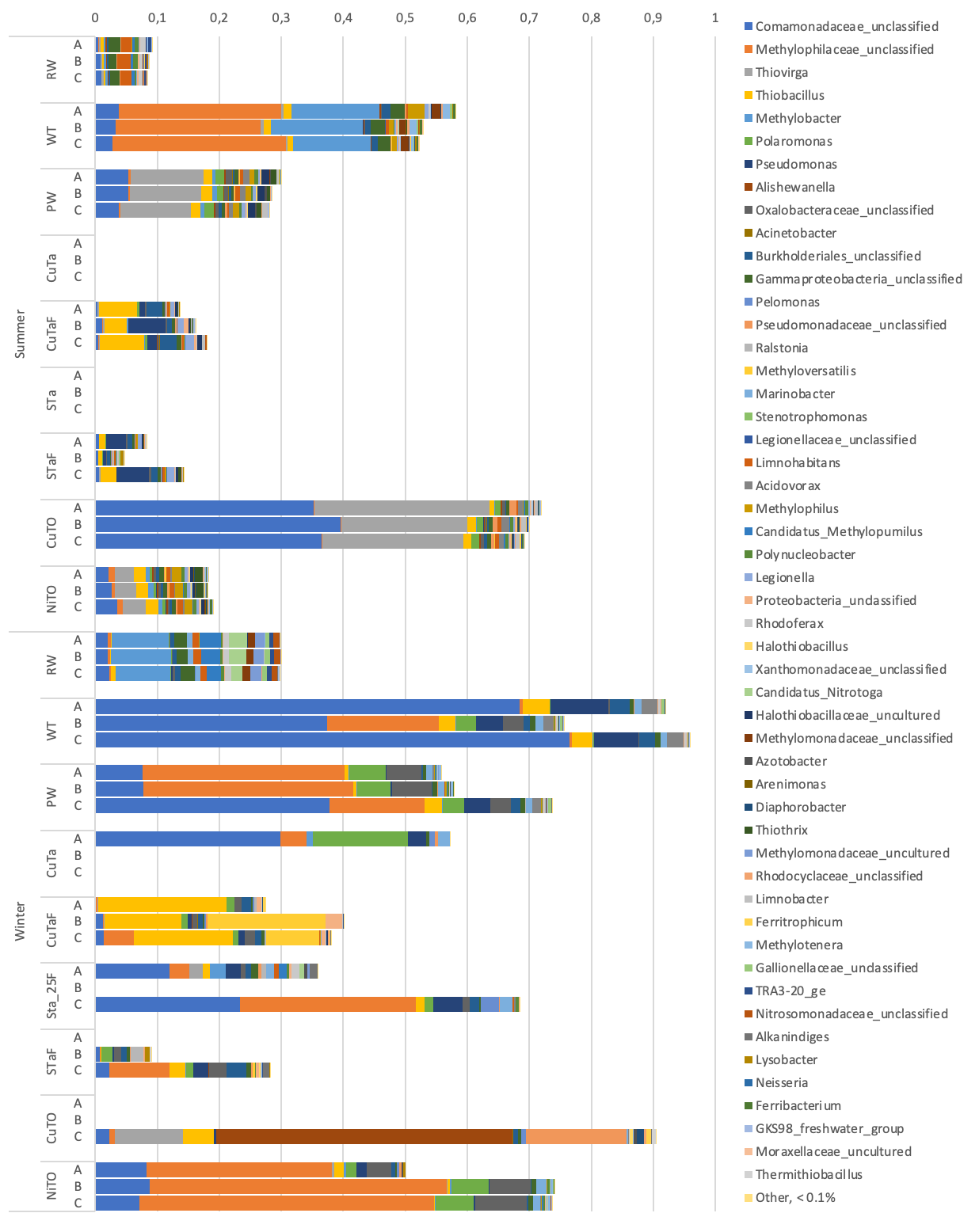
**Figure SA3.** The relative abundances of gammaproteobacterial lineages identified from the summer and winter samples by amplicon sequencing of the bacterial 16S rRNA genes. The ‘Other’ category sums up all gammaproteobacterial taxa representing less than 0.1% of the bacterial community in all samples.


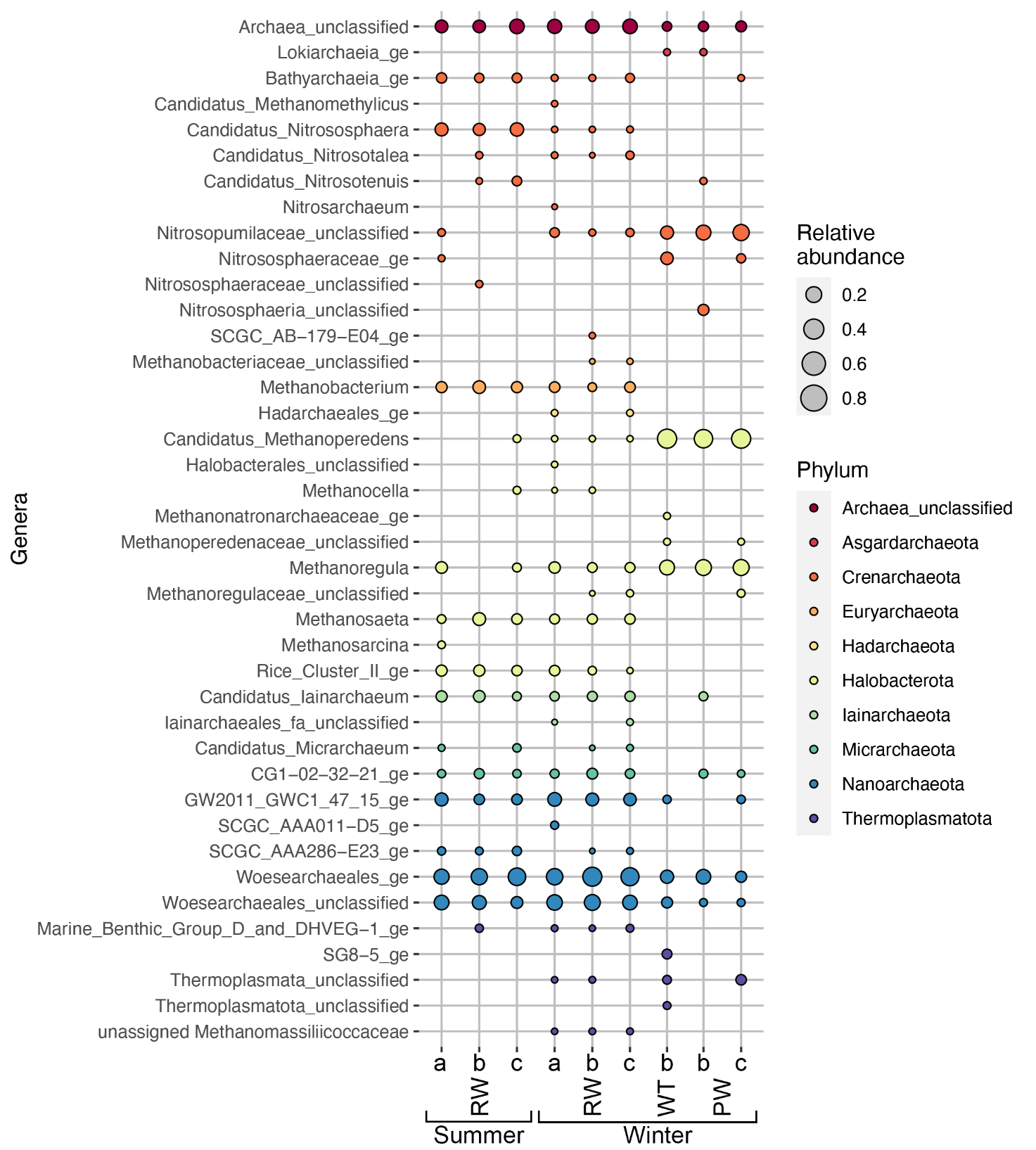


**Figure SA4.** The relative abundance of the 50 most prominent archaeal genera per sample. “Other” indicate all other less abundant genera.


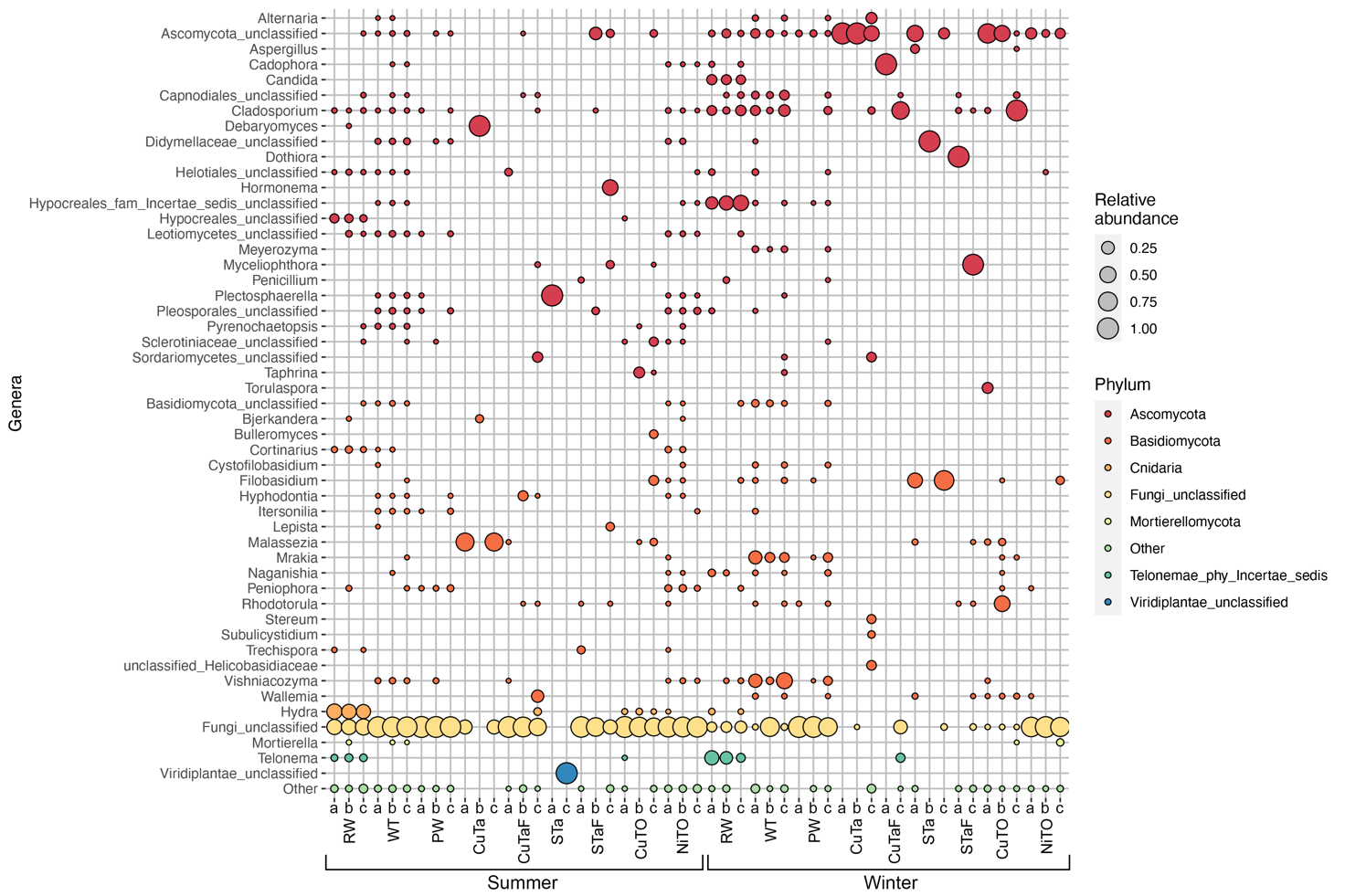


**Figure SA5.** The relative abundance of the 50 most prominent eukaryotic genera in the individual samples. “Other” indicate all other less abundant genera.

**References**

1. Andrews, S. (2010). FastQC: a quality control tool for high throughput sequence data. <http://www.bioinformatics.babraham.ac.uk/projects/fastqc>
2. St John, J. (2011). SeqPrep: Tool for stripping adaptors and/or merging paired reads with overlap into single reads. [*https://githubcom/jstjohn/SeqPrep*](https://githubcom/jstjohn/SeqPrep)
3. Bolger, A.M., Lohse, M. and Usadel, B. (2014). Trimmomatic: a flexible trimmer for Illumina sequence data. *Bioinformatics*. *30*(15). pp.2114-2120.
4. Eren, A.M., Esen, Ö.C., Quince, C., Vineis, J.H., Morrison, H.G., Sogin, M.L. and Delmont, T.O. (2015). Anvi’o: an advanced analysis and visualization platform for ‘omics data. *PeerJ*. *3*. p.e1319.
5. Li, D., Liu, C. M., Luo, R., Sadakane, K., & Lam, T. W. (2015). MEGAHIT: an ultra-fast single-node solution for large and complex metagenomics assembly via succinct de Bruijn graph. *Bioinformatics*, *31*(10), 1674-1676.
6. Mikheenko, A., Saveliev, V., & Gurevich, A. (2016). MetaQUAST: evaluation of metagenome assemblies. *Bioinformatics*, *32*(7), 1088-1090.
7. Langdon, W.B. (2015). Performance of genetic programming optimised Bowtie2 on genome comparison and analytic testing (GCAT) benchmarks. *BioData mining*. *8*(1). p.1.
8. Menzel, P., Ng, K. L., & Krogh, A. (2016). Fast and sensitive taxonomic classification for metagenomics with Kaiju. Nat Commun 7: 11257.
9. Ondov, B. D., Bergman, N. H., & Phillippy, A. M. (2011). Interactive metagenomic visualization in a Web browser. *BMC bioinformatics*, *12*(1), 1-10.
10. El-Gebali, S., Mistry, J., Bateman, A., Eddy, S. R., Luciani, A., Potter, S. C., ... & Finn, R. D. (2019). The Pfam protein families database in 2019. *Nucleic acids research*, *47*(D1), D427-D432.
11. Seemann, T. (2014). Prokka: rapid prokaryotic genome annotation. *Bioinformatics*. *30*(14). pp.2068-2069.
12. Kanehisa, M., Sato, Y. and Morishima, K. (2016). BlastKOALA and GhostKOALA: KEGG tools for functional characterization of genome and metagenome sequences. *Journal of molecular biology*. *428*(4). pp.726-731.
